# The impact of the COVID-19 pandemic and associated lifestyle changes on early-life microbiome development

**DOI:** 10.1186/s13073-026-01660-8

**Published:** 2026-04-29

**Authors:** Evgenia Dikareva, Niels van Best, Liene Bervoets, Christina E. West, Connor Rossel, Christel Driessen, Monique Mommers, John Penders

**Affiliations:** 1 Department of Medical Microbiology, Infectious Diseases and Infection Prevention, Institute of Nutrition and Translational Research in Metabolism (NUTRIM), Maastricht University Medical Centre+, PO5800, Maastricht, 6202 AZ The Netherlands; 2 Institute of Medical Microbiology, RWTH Aachen University Hospital, Aachen, Germany; 3 Department of Clinical Sciences, Pediatrics, Umeå University, Umeå, Sweden; 4 Department of Epidemiology, Care and Public Health Research Institute (CAPHRI), Maastricht University, PO 616, Maastricht, 6200 MD The Netherlands

**Keywords:** COVID-19, Pandemic, Hygiene, Microbiome, Social distancing

## Abstract

**Background:**

The COVID-19 pandemic triggered rapid, population-wide behavioral and environmental changes, offering a unique natural experiment to study how early-life microbiome development responds to abrupt shifts in social and hygiene-related exposures.

**Methods:**

Using longitudinal data from 139 infants in the Dutch LucKi Gut study, we compared gut microbiome development in fecal samples collected before and during the pandemic. Whole metagenome sequencing of 808 stool samples was performed across nine time points in the first 14 months of life. An exposure index (EI) capturing variation in household-level pandemic-related behaviors was constructed for the 36 infants with samples collected during the COVID-pandemic to quantify variations in social distancing, lifestyle and hygiene measures.

**Results:**

Microbial richness and diversity increased with age, following established developmental trajectories. However, from 6 months onward, the COVID-19 pandemic independently shaped gut microbial composition, explaining up to 2.7% of variation by 11 months of age (Q-value = 0.006). Forty-four species were differentially abundant in pandemic-era samples, including depletion of *Gordonibacter pamelaeae* and several *Actinomyces* species. Notably, greater environmental exposure (higher EI scores) was associated with lower abundance of *G. pamelaeae*, a microbe implicated in bile acid and immunomodulatory metabolism.

**Conclusions:**

This is the first longitudinal whole-genome sequencing study to demonstrate that pandemic-related behavioral changes measurably altered infant gut microbiota maturation. These findings highlight the sensitivity of microbiome development to societal-level environmental disruptions and suggest that early-life microbial exposures, modulated by hygiene and social behavior, may carry long-term implications for child health.

**Supplementary Information:**

The online version contains supplementary material available at 10.1186/s13073-026-01660-8.

## Background

An increasing number of studies emphasize the significance of the gut microbiome in infant health. The early-life gut microbiome plays a crucial role in shaping long-term health by influencing immune development, host metabolism, and disease susceptibility [1–7]. The establishment of the microbiome during childhood follows a dynamic progression that can be divided into three phases: a developmental phase, a transition phase, and a stable phase [8]. This process is largely orchestrated by microbial transmission from the mother, family members, and the environment while genetic factors, infant feeding practices, and dietary transitions also play important roles [9].

However, while breastfeeding and complementary feeding are key determinants of microbiota composition [8, 10–13], they account for only a fraction of the interindividual variation in microbial community structure [14]. Given the established links between early-life microbiota and the risk of allergic and autoimmune diseases [15–18], identifying additional factors that shape or disrupt microbiota establishment is essential. The "hygiene hypothesis" proposes that reduced exposure to infections contributes to the rise in non-communicable diseases [19, 20], whereas the "old friends hypothesis" suggests that evolutionary shifts in microbial exposure, rather than a lack of infections per se, underlie these trends [21, 22].

Although changes in social interactions, hygiene practices, and lifestyle typically occur gradually over time, the onset of the COVID-19 pandemic led to abrupt and widespread behavioral and environmental shifts [23–25]. This unprecedented global event presents a unique opportunity to investigate how such factors influence gut microbiome development in early life.

During the pandemic, the incidence of infectious diseases—including gastrointestinal infections—and antibiotic prescriptions for respiratory infections declined significantly in the Netherlands [26, 27], largely due to social distancing measures and daycare closures [28]. Additionally, lifestyle changes varied across the population, with some individuals adopting healthier dietary and physical activity habits, while others exhibited the opposite trend [25, 29].

These pandemic-induced behavioral shifts have the potential to alter infant gut microbiota development. A study of 12-month-old American infants reported reduced alpha diversity and decreased abundances of Pasteurellaceae and *Haemophilus* in pandemic-era samples compared to pre-pandemic cohorts [30]. Similarly, the CORAL study in Ireland found that pandemic-era infants had increased *Bifidobacterium* levels and decreased *Clostridium* abundance, possibly due to reduced exposure to individuals outside the household [31]. Comparable findings were observed in a Chinese cohort, where pandemic-born infants exhibited reduced microbial diversity, altered community composition, and decreased antimicrobial resistance gene carriage [32].

However, these studies either analyzed microbiota at a single time point [30] or lacked a pre-pandemic comparison within the same cohort [31, 32], limiting their ability to assess longitudinal microbiota development across pandemic and non-pandemic periods in the same population.

Here we study the impact of the COVID-19 pandemic on gut microbiota establishment during infancy within a single cohort, the Dutch Lucki Gut study. This longitudinal birth cohort was designed to track the longitudinal development of the infant gut microbiome, began recruitment in 2016 and continued enrolling participants throughout the pandemic. This provided a unique opportunity to examine the impact of the COVID-19 pandemic on gut microbiota establishment within a single cohort. We hypothesized that lockdown and strict hygiene measures would have an impact on the infants’ gut microbiota development. To test this, we performed whole metagenome sequencing on samples collected pre and during the COVID-19 pandemic. The resolution of the sequencing allowed us to examine differences at species level.

## Methods

### Study population and sample collection

The LucKi Gut study is an on-going longitudinal study among newborns and their families. Pregnant women residing in the South Limburg region of the Netherlands were recruited through obstetrics and gynaecology clinics, lactation information sessions, and advertisements at pregnancy yoga classes, baby clothing stores, and on social media. Infants born prematurely (gestational age ≤ 32 weeks) were excluded. Infant fecal samples were collected at 1–2 weeks post-partum and again at 1, 2, 4, 5, 6, 9, 11 and 14 months of age (Additional file 1: Table S1A). These time-points align with the ages at which children have scheduled visits to well-baby clinics.

Participants received fecal sampling starter kits consisting of stool collection tubes (Sarstedt, REF 80.623.022), cold transport containers (Sarstedt, REF 95.1123), a safety bag, gloves, questionnaires, and instructions. The samples were collected at home and immediately stored at −20°C in their home freezers. Samples were thereafter transported to the family’s well-baby clinic using a frozen transport container to preserve the cold chain. From there, samples were transported to the laboratory, where frozen fecal matter was aliquoted and stored at −80°C until further analyses.

At each fecal sampling time-point, parents also completed a questionnaire gathering information on the infant's lifestyle, health, development, medication use, and feeding practices, as well as maternal health (during pregnancy), diet, and medication use. Alongside the questionnaires we collected 808 fecal samples from 139 infants recruited between August 2016 and November 2022.

After the onset of COVID-19 pandemic, the newly enrolled families and families that were still in follow-up (*n* = 36) also filled in a separate questionnaire on social distancing, protective measures, hygiene and other pandemic related measures (Additional file 1: Table S1B). Informed consent was provided by all parents. The study was approved by the medical ethics committee of the Maastricht University Hospital (METC 15–4–237).

### Metadata processing

Information on perinatal determinants, lifestyle, diet, medication use, and health outcomes was collected through self-reported questionnaires collected around birth (pregnancy questionnaire and paternal and maternal questionnaires) and at each subsequent sampling time point.

The following variables were included for the purpose of the present study: gestational age (weeks), birthweight (grams), maternal weight gain (kilograms), age at complementary food introduction (weeks), delivery type (C-section, vaginal), maternal atopy (no, yes), paternal atopy (no, yes), infant antibiotic use since previous follow-up moment (no, yes), breastfeeding since previous follow-up moment (no, yes), formula feeding since previous follow-up moment (no, yes), day care attendance (no, yes), maternal smoking before pregnancy (no, yes), older siblings (no, yes), siblings in the cohort (no, yes), furry pets at home (no, yes), sex (male, female), delivery place (hospital, at home), infant hospital admission upon birth (no, yes), maternal antibiotics during delivery (no, yes) and maternal antibiotics during pregnancy (no, yes) (Additional file 1: Table S2A). For the follow-up time points where less than 5% of the children were reported to have used antibiotics, the variable on antibiotic use was omitted from the analyses. Missing information for breast and formula feeding was imputed as follows: if information on infant feeding was available and identical at both the preceding and subsequent time-point, then this value was imputed for the intermediate time-points. If breast milk was not given at the first time-points (1–2 and 4 weeks), then “no breastfeeding” was imputed for subsequent time-points for which information was missing. Otherwise, missing values were not imputed. For data on furry pets missing values were imputed as follows: if the information was available at two times-points (at birth and 14 months, or at birth and 6 months) and it was identical, then this value was imputed for the missing time-point. Otherwise, missing values were not imputed. For missing numeric values (gestational age, birthweight, maternal weight gain and age of complementary food introduction), the mean was calculated and used to impute missing values (Additional file 1: Table S2A). The frequencies of the variables for each time point can be found in Additional file 1: Table S3A.

To assess the diversity of the infants’ complementary food, various scores were calculated for each time point per infant. Food Variety Score (FVS) [33] reflects the number of unique food items introduced to the infant between 4–14 months (maximum of 28). Dietary Diversity Score (DDS) [33] reflects the number of unique food groups introduced. To this end, food items were grouped into eight groups representing vegetables, fruits, legumes/nuts, meat, fish, eggs, dairy, and grains as was described previously.

Descriptive statistics for infants of whom all samples were collected prior to the pandemic infants as compared to infants of whom (most) samples were collected during the pandemic are summarised in Additional file 1: Tables S2B and S3B-3C.

The “*pandemic*” variable categorized samples into two groups based on their collection date relative to the onset of the COVID-19 pandemic in the Netherlands. Samples collected before February 27, 2020, (the date of the first confirmed infection by the SARS-CoV-2 virus in the Netherlands) were assigned to the "pre-pandemic" group, while samples collected on or after this date were assigned to the "pandemic" group (Additional file 1: Table S1B).

For the subgroup of 36 families in which at least one fecal sample was collected during the COVID-19 pandemic, we additionally created an “*exposure index*” (EI) to estimate the level of social interactions, lifestyle and hygiene measures. To this end, we initially selected 58 questions related to the COVID-19 pandemic, excluding those related to SARS-CoV-2 infections directly such as diagnostic testing and symptoms. For highly correlated variables (Pearson’s rho > 0.8) one of the variables was removed and remaining variables were further processed. From these individual variables (Additional file 1: Table S4) we created new variables that aggregated the level of exposure (Additional file 2: Fig. S1, Additional file 1: Table S5). For example, for variables on exposure in indoor public spaces, the number of days per week people had social interactions in public space, the number of times they kept distance and/or were wearing a mask during such social interactions were combined into one new variable that provides a summed score with the highest value for individuals with frequent unprotected interactions in indoor public spaces. All variables were coded in such a way that a higher score was related to more exposure (Additional file 1: Table S4).

Next, we rescaled these newly created variables according to the median for the answers (0, when original value was 0, 1 when below median and 2 when above or equal to the median). Binary variables were kept as binary variables. Finally, these rescaled variables were summed into the “exposure index” (EI).

Two versions of the EI were initially constructed: one incorporating responses related to social interactions, hygiene practices, parental occupation, and protective measures used by both parents, and another excluding paternal (or second parent) responses. Given the high level of correlation (Spearman’s ρ = 0.940, *p*-value < 0.001) between both indices (Additional file 2: Fig. S2), we ultimately continued with the latter index since information on questions related to the father (or second parent) was only available for 32 out of the 36 families.

Due to a rapid surge in COVID-19 cases soon after the first case on February 27th, 2020, strict hygiene recommendations and social-distancing measures were implemented very early in the pandemic. The full timeline of actions performed by the Dutch government can be found in Additional file 1: Table S6. For the subgroup of the samples collected during the pandemic, we calculated dichotomized time spent in pandemic. First the difference in days between the sample collection date and the start of the pandemic in the Netherlands (February 27th, 2020) was calculated. The median of the calculated time difference in days was determined for each age. Each sample was subsequently categorized as below or equal to the median vs. above the median. This dichotomized variable was used in further sensitivity analysis.

### DNA isolation, whole metagenomic sequencing and data pre-processing

Approximately 100 mg of aliquoted fecal sample, was sent to MGI Tech Latvia (Mārupe, Latvia) for DNA extraction and whole metagenomic sequencing. Metagenomic DNA was isolated using the MagPure Stool DNA LQ kit according to the manufacturer’s protocol (Magin Biotech, Guangzhou, China), with the additional inclusion of a mechanical bead-beating step using 0.1 mm glass beads as described previously [34]. Library preparation and shotgun metagenomic sequencing were performed on the BGISEQ-500 platform using the paired-end 150 mode.

Pre-processing of sequencing reads was performed according to the "remove-host" standard operating procedure in MMHP (the Million Microbiome of Humans Project). To standardize the pipeline a workflow manager Snakemake v.5.14.0 [35] was used. Quality filtering was performed using Fastp v.0.20.1 with default quality threshold of Q15, a minimum read length of 60 bp, and rejection of any reads containing N bases [36]. The same tool was used to trim the BGI-SEQ adapters [37] “AAGTCGGAGGCCAAGCGGTCTTAGGAAGACAA” for forward and “AAGTCGGATCGTAGCCATGTCGTTCTGTGAGCCAAGGAGTTG” for reverse reads. Human reads were removed by mapping against the Telomere-to-telomere consortium CHM13 human reference genome (v.1.0, including the hg38 Y chromosome; downloaded 14.10.2020) [38] using Bowtie 2 v.2.3.5.1 with the very-sensitive preset and a maximum paired-end alignment length of 600 bp [38, 39]. Paired reads where both mates failed to align were retained using Samtools v.1.9 (-f 12 -F 256) [40]. Taxonomic composition was determined using MetaPhlAn v.3.0 [41] with default parameters (Bowtie2 very-sensitive preset, stat_q = 0.2, minimum clade marker length = 2000 bp, minimum read length = 70 bp) and the mpa_v30_CHOCOPhlAn_201901 marker gene database [41].

### Statistical analysis and data visualization

Statistical analysis and data visualisation were performed on Rstudio (v.2023.06.2 + 561) with integrated R (v.4.1.3) [42]. With the phyloseq (v.1.38.0) [43] and tidyverse (v.2.0.0) [44] packages the phyloseq object was constructed. For data visualisation microViz (v.0.10.8) [45] and viridis (v.0.6.2) [46] packages were used.

To reduce the sparsity of the data, bacterial taxa were filtered out at species level with a prevalence of < 5% (microViz, tax_filter(min_prevalence = 0.05, tax_level = "Species") across all samples. These filtered data were used in all downstream analysis except for alpha diversity analysis where unfiltered profiles were used.

#### Alpha diversity, univariable and multivariable analysis

The following ecological diversity distances were calculated with vegan package (v.2.6–4) [47]: Shannon index and observed richness. The Effective Number of Species (ENS) was subsequently calculated from the Shannon index using the base R exponential function (exp()) [48].

Normality of alpha diversity indices was assessed using the Shapiro–Wilk test and visual inspection (histograms). Because ENS and observed richness deviated from normality, alpha diversity is summarized as median (IQR) and paired Wilcoxon signed-rank tests were used to compare indices between subsequent time points. (p.adjust.method = "BH", alternative = "two.sided", paired = T). The *p*-values were adjusted with false discovery rate (FDR) correction (Q-values) using the Benjamini–Hochberg (BH) procedure [49] with function p.adjust from stats package (v.4.1.3) for each alpha diversity metric separately.

To analyse whether variables were associated with alpha diversity, linear regression analysis was performed for each time point using function lm() from *stats* package. The numeric variables were scaled using the R basic function scale(). Model assumptions were assessed using regression diagnostics, including Q–Q plots of standardized residuals and residuals-versus-fitted plots to evaluate approximate normality and homoscedasticity of residuals. Despite non-normality of the raw alpha diversity distributions, residual diagnostics indicated that the linear model assumptions were sufficiently met for inference at each time point. Backward elimination was performed by iteratively removing variables with *p*-value > 0.20.

#### Principal component analysis and beta diversity evaluation

The Aitchison’s distance [50] was used to analyse beta-diversity and overall variation between the samples. Ordination of infant fecal samples was performed by principal component analysis (PCA) using microViz package. The reads were center log ratio (CLR) transformed on “Species” level (tax_transform("clr", rank = "Species")) upon which samples were arranged by similarity into new dimensions to form PCA (ord_calc(method = "PCA")). To visualize the species composition of samples, a circular compositional barplot (IRIS plot) sorted by the PCA ordination angle was created (microViz, tax_transform("clr", rank = "Species")). A permutational multivariate analysis of variance (PERMANOVA) was conducted using the pairwise.adonis() function from vegan package to assess differences between the ages. *P*-values were FDR adjusted for final models for all time points at a time. The Skillings–Mack test was used to compare Aitchison distance between the time points with a Wilcoxon paired signed rank test as a post hoc test.

#### Dirichlet multinomial mixture clustering

Dirichlet Multinomial Mixture (DMM) clustering was performed using the Dirichlet Multinomial package (v.1.36.0) at the species level. Samples were assigned to a specific cluster according to the Laplace approximation score, which represents a specific cluster or a signature composition of microbes. The clustering procedure [51] and transition analyses [8] were conducted as previously described. Species-level profiles were CLR-transformed and analyzed by PCA. Samples were plotted and coloured by DMM cluster, with dashed 95% t-distribution confidence ellipses shown for each cluster.

#### Marginal permutational multivariate analysis of variance

To identify variables that were significantly associated with the microbial community structure, a Marginal PERMANOVA based on marginal sum of squares was performed with microViz. PERMANOVA was conducted on filtered reads with 999 permutations. For each time point, Aitchison distance matrices were calculated on species-level using microViz. *P*-values were FDR corrected across all the time points at once with p.adjust() function from package stats.

For the first three time points (1, 4, and 8 weeks), the following variables were selected: parental atopy, birthweight, breastfeeding and formula feeding, delivery place and type, hospital admission upon delivery, maternal antibiotic use during pregnancy and delivery, maternal weight gain during pregnancy, older siblings, presence of siblings inside the cohort, gestational age, sex, maternal smoking before pregnancy and the pandemic variable.

For the remaining time points, the following additional variables were added: age at the introduction of complementary foods and daycare attendance. Only the last three time points had enough observations (> 5% of “yes” answers) for infant antibiotic exposure since previous sampling time point.

For each time point, a series of PERMANOVAs were performed, and the backward elimination method was implemented. Variables with the highest *p*-value were removed iteratively until only those variables with *p* ≤ 0.200 remained. For each time point, a distinct set of variables was retained in the final model. These were used in further analysis and are referred to as the 'final set of variables' for each respective time point.

#### Sensitivity analysis

To ensure that the impact of the pandemic on the infant microbiome was not a technical artefact related to sequencing batch effects, we ran a sensitivity analysis including the sequencing batch as an additional covariate. This could however only be performed in case samples were sufficiently spread across sequencing batches (e.g., not for 9 and 11 months of age). Sensitivity analyses revealed that while technical variation significantly explained up to 5.6% of interindividual variation in microbiome profiles, it did not eliminate the effect of the pandemic at 6 months (Q-value = 0.046) and as such rules out that observed findings were due to technical artefacts (Additional file 2: Fig. S3, Additional file 1: Table S7). As older siblings are a known source of microbial and social exposures and may buffer reductions in extra-household contacts during the pandemic, we repeated the main analyses in the full study population but stratified for the presence of older siblings. Specifically, for each sampling age we (i) assessed differences in overall microbial community structure between pre-pandemic and pandemic samples using PERMANOVA on the beta-diversity distance matrix and (ii) examined differences in microbial richness using linear regression models. All models used the same outcome definitions, covariate adjustments, and multiple-testing correction approach as in the primary analyses. Results are reported as stratum-specific effect estimates and explained variance (PERMANOVA R^2^) with corresponding FDR-adjusted q-values.

To explore whether microbiome differences were related to duration of exposure to pandemic conditions, we calculated the time elapsed (in days) between 27 February 2020 (first confirmed SARS-CoV-2 infection in the Netherlands) and the date of sample collection for each sample. For each sampling age, pandemic samples were stratified into “early” vs “late” pandemic collection (below or equal to vs above the median elapsed time). Next, the main analyses were repeated within the subgroup of samples collected within the pandemic with duration of exposure to pandemic conditions as explanatory variable. Owing to limited sample size, these sensitivity analyses were restricted to (i) PERMANOVA to assess associations with community structure and (ii) models of alpha diversity (microbial richness), applying the same covariate adjustments and multiple-testing correction as in the primary analyses.

#### Differential abundance analysis

To detect bacterial species that were significantly different in abundance between time points, a linear model for differential abundance analysis (LinDa) was performed using the LinDa package (v.0.1.0) [52] (alpha = 0.2, prev.cut = 0, lib.cut = 1, winsor.quan = NULL, corr.cut = 0.1, p.adj.method = 'BH', type = "count"). Separate models were run for each follow-up time with default settings with the “final set of variables”. To reduce the false discovery rate, the *p*-values were adjusted using the BH procedure (Q-value) within each time point, and only Q-values ≤ 0.2 [53] are presented. The relative taxon abundances were visualised in heatmaps presenting the log fold differences in bacterial abundance.

#### Impact of exposure index on alpha and beta diversity and abundance of individual species

To investigate the effect of the EI for the 5–14-month time points, we used samples collected after the onset of the pandemic. The EI variable was used in place of the original pandemic variable. For the 6-month time point, the variable "delivery place" was excluded due to collinearity with the "siblings in cohort" variable. The impact of EI on alpha diversity was assessed as previously described for ENS and observed richness. Beta diversity was analysed using PCA plots and PERMANOVA, as outlined above. Differential abundance analysis was also performed as described previously.

### Language editing

ChatGPT (OpenAI) was used to assist with language editing and improving grammar in this manuscript. The authors take full responsibility for the content and interpretation of the results.

## Results

**Fig. 1:**
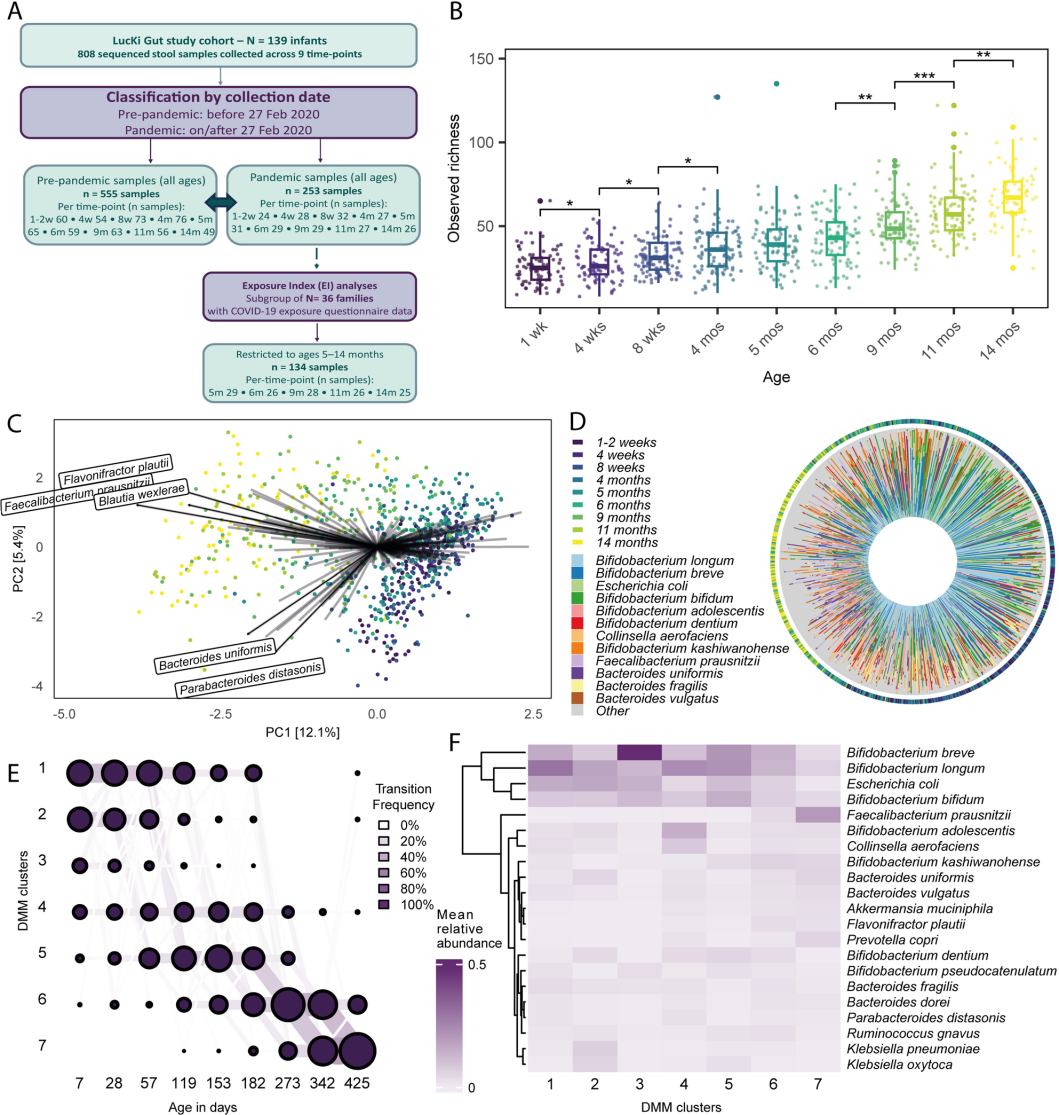
Temporal dynamics and taxonomic composition of infant microbiome. **A** Flow diagram of included samples. The full cohort comprised 139 infants contributing 808 sequenced stool samples across nine scheduled time points. Samples were categorized as pre-pandemic (before 27 Feb 2020; n = 555) or pandemic (on/after 27 Feb 2020; n = 253). Exposure Index (EI) analyses were conducted in a subgroup of 36 families with COVID-19 exposure questionnaire data and included only pandemic-era samples collected at 5–14 months (total n = 134). **B** Changes in observed richness over time. Individual samples are displayed as jittered points. The FDR adjusted *p*-values are represented with stars, where “***” indicates Q-values < 0.001, “**” indicates Q-values between 0.001 and 0.01, and “*” indicates Q-values between 0.01 and 0.05. **C** Beta-diversity at different time points. The PCA is based on centered log-ratio (clr)-transformed reads at the species level. The arrows represent taxon loading vectors that drive the majority of the variation. **D** Iris plot displaying the top 12 abundant species. The samples are arranged in the same sequence as in the PCA plot. **E** Infants' microbiota transition across Dirichlet Multinomial Mixture (DMM) clusters over time. The thickness of the lines represents transition frequency, and the size of the nodes represents the number of infants in each particular cluster at each time point (in days). **F** Heatmap displaying the mean relative abundance of the most dominant bacterial species for each DMM cluster. Only 21 bacteria species with a relative abundance > 0.01 are presented

A total of 808 fecal samples collected from 139 infants at the ages of 1–2, 4 and 8 weeks, 4, 5, 6, 9, 11 and 14 months were successfully sequenced (Additional file 1: Table S1A). Out of the 808 samples, 253 (31.3%) were collected during the COVID-19 pandemic (Fig. 1A, Additional file 1: Table S1A). Additional questionnaire data on societal interactions, lifestyle and hygiene measures during the pandemic were collected for the 36 participating families in which at least one stool sample from the child was collected during the COVID-19 pandemic (Additional file 1: Table S1B).

Of the 139 included children, 80 (57.6%) were boys, 117 were born vaginally (84.2%) and 121 were born in the hospital (87.1%). Moreover, 59 (42.2%) infants had older siblings (Additional file 1: Table S2A). Breastfeeding was initially given to most infants (88.1%) during the first weeks of life and over half (52.3%) of the infants were still being breastfed by 6 months of age. Antibiotic exposure during the first 6 months of life was rare with only 1 to 4 infants exposed prior to each of the sampling time-points (Additional file 1: Table S3A). By the age of 18 weeks, complementary foods had been introduced to half of the infants (median: 18 weeks, Interquartile range (IQR): 4.0 weeks, Additional file 1: Table S2A). Neither the age of introduction of solid foods, nor the diversity of food items or food groups introduced differed between prepandemic and pandemic subgroups of infants (Additional file 1: Tables S2B, S3C).

Infants born and longitudinally sampled before the COVID-19 pandemic had similar demographic and lifestyle characteristics as compared to infants whose longitudinal sampling largely overlapped with the pandemic (Additional file 1: Table S2B and S3B) except for a lower proportion of maternal antibiotic use during pregnancy (9.4% vs. 23.3%) and higher proportion of infant antibiotic use at 9 months (20.6% vs. 3.4%) and 11 months (10.7% vs. 0%) among pre-pandemic infants.

### Temporal changes in the infant microbiome: microbial richness and diversity increase over time, with the infant gut being dominated by *Bifidobacterium* species

In line with previous studies [54–57], we observed an increase in microbial richness and diversity with age. Observed richness was statistically significantly different between the ages 1 and 4 weeks (Wilcoxon rank-sum test, W = 703, Q-value (FDR) = 0.049); 4 and 8 weeks (W = 885, Q-value = 0.049); 8 weeks and 4 months (W = 1124, Q-value = 0.049); 6 and 9 months (W = 570, Q-value = 0.002); 9 and 11 months (W = 476, Q-value < 0.001) and 11 and 14 months (W = 503, Q-value = 0.004, Fig. 1B, Additional file 1: Table S8). The difference for Effective Number of Species (ENS) was significant between 6 and 9 months (W = 645, Q-value = 0.01) and 9 and 11 months (W = 786, Q-value = 0.03, Additional file 2: Fig. S4, Additional file 1: Table S8).

Age was also the primary factor explaining variation in microbial community structure, as evidenced by the separation of samples in the PCA plot (Fig. 1C). A Skillings–Mack test used to compared Aitchison distance between the time points indicated a statistically significant difference between the ages (statistics = 185.82*,* p < 0.001). The most pronounced shift in microbial community composition occurred between 6 and 9 months of age (p < 0.001), as reflected by the increased Aitchison distances between adjacent time points (Additional file 2: Fig. S5, Additional file 1: Table S9).

Across all time points, *Bifidobacterium* species, such as *B. longum* and *B. breve,* along with *Escherichia coli*, were among the most abundant species. At later time points, various *Bacteroides* species, *Collinsella aerofaciens*, *Phocaeicola vulgatus* (previous name—*Bacteroides vulgatus* [58]), and *Faecalibacterium prausnitzii* increased in relative abundance (Fig. 1D).

We next applied Dirichlet multinomial mixtures (DMM) modelling to cluster bacterial communities over time (Fig. 1E). Each cluster was defined by distinct microbial compositions (Fig. 1F, Additional file 1: Table S10), reflecting progressive microbiome maturation (Additional file 2: Fig. S6). Most infants initially belonged to cluster 1, dominated by *B. longum*, *B. breve*, and *E. coli*, and transitioned to cluster 5, characterised by dominance of *B. longum*, *B. breve*, and *Bifidobacterium bifidum and* reduced *E. coli*—by 4 months of age. By 9 months, most had shifted to cluster 6, followed by further transition to cluster 7 in later months, marked by the emergence of *F. prausnitzii*.

### The COVID-19 pandemic affected microbial diversity and composition

**Fig. 2:**
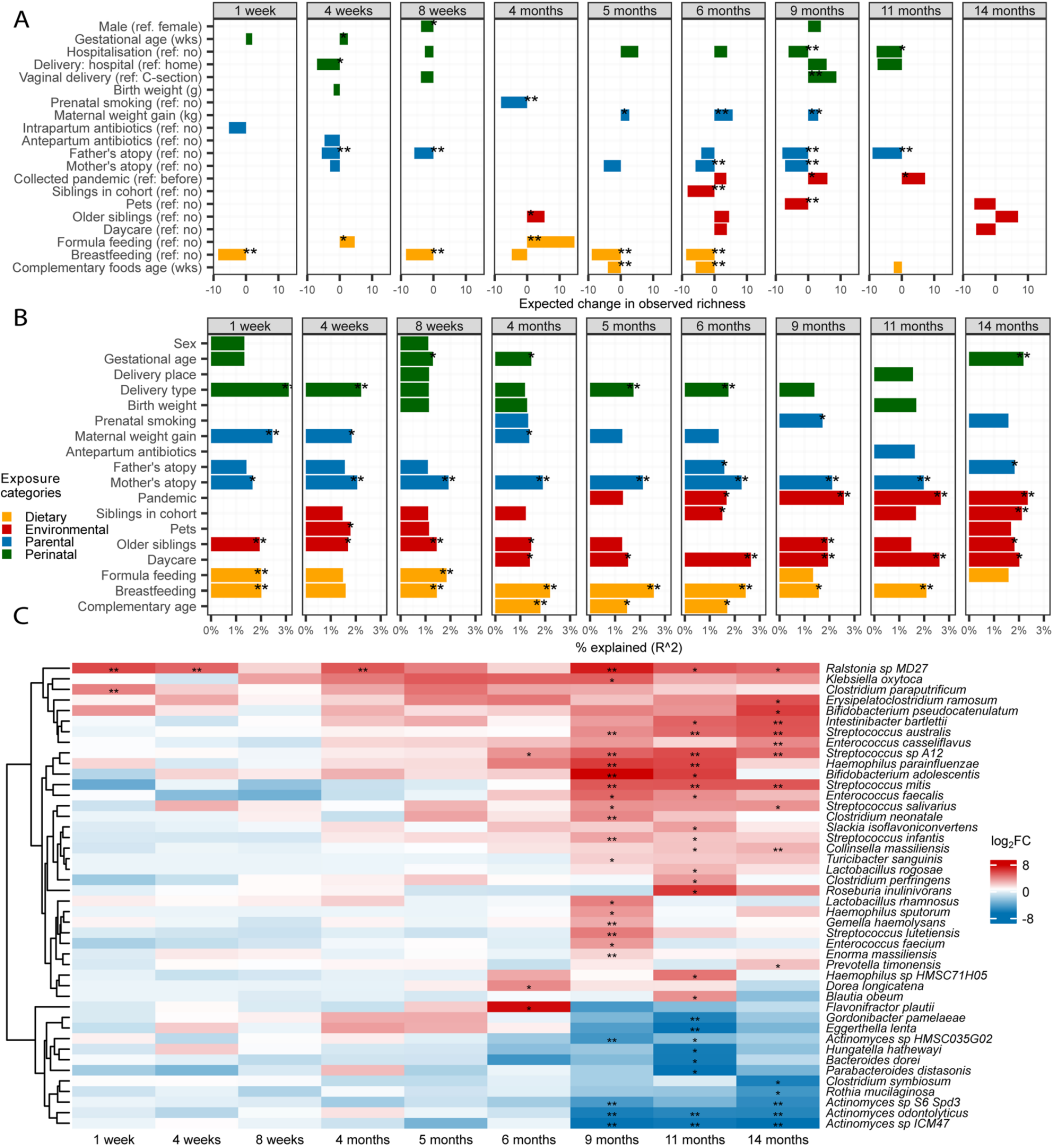
COVID-19 pandemic is one of the determinants affecting microbial composition, diversity and species abundance. **A** Bar charts depicting the regression coefficients of each variable in association to the observed richness at each time point. Significant Q-values are indicated with stars: “**” indicates Q-values < 0.1, “**” indicates Q-values < 0.05. **B** Variance explained (R^2^) by each variable per time point (PERMANOVA on Aitchison distances). Significant Q-values are indicated with stars: “**” indicates Q-values < 0.1, “**” indicates Q-values < 0.05. **C** Heat map of the 44 bacterial species which showed statistically significant differences in abundance in pandemic as compared to pre-pandemic samples across each time point (LinDa). The number of pre-pandemic and pandemic samples included at each time-point is as follows, 1 week: 60 vs. 24, 4 weeks: 54 vs. 28, 8 weeks: 73 vs. 32, 4 months 76 vs. 27, 5 months: 65 vs. 31, 6 months: 59 vs. 29, 9 months: 63 vs. 29, 11 months: 56 vs. 27, 14 months: 49 vs. 26. The color gradient ranges from blue (lower abundance) to red (higher abundance), representing log changes. Statistically significantly differentially abundant species are indicated with stars: “**” indicates Q-values < 0.05 and “*” for Q-values between 0.05 and 0.2. Log_2_ FC: Log_2_ Fold Change

To understand the effect of various variables on infants’ gut microbial richness and diversity, we performed linear regressions on alpha diversity metrics. The observed microbial richness was most strongly influenced by infant feeding, with breastfeeding being associated with a lower and formula feeding with a higher microbial richness at several time-points during the first 6 months of life (Fig. 2A, Additional file 1: Table S11). Maternal and paternal atopy was associated with a lower microbial richness in the infant microbiome, and early complementary food introduction was associated with an increase in microbial richness. Besides these well-known drivers of microbial richness, the COVID-19 pandemic was associated with a statistically significant increase in observed richness at 9 months of age (estimate = 5.9, 95% CI [0.7, 11.2], Q-value = 0.055) (Fig. 2A, Additional file 1: Table S11). Moreover, the fecal microbial diversity was marginally higher in 11-month-old infants sampled during the pandemic as compared to infants sampled prior to the pandemic (estimate = 1.8, 95% CI [−0.02, 3.7], Q-value = 0.091, Additional file 2: Fig. S7, Additional file 1: Table S11).

Next, we examined factors influencing the gut microbial community structure. We identified multiple perinatal, environmental, dietary, and health-related determinants (Fig. 2B, Additional file 1: Table S12) contributing to the inter-individual variation in the gut microbiota composition. Mode of delivery was a major determinant, exerting its strongest influence between 1 and 2 weeks of age and explaining over 3% of the variation (Q-value = 0.004), with effects persisting up to 6 months (R2 = 1.7%, Q-value = 0.048). Maternal atopy (R2: 1.7–2.3%, all Q-value < 0.05 except 1–2 weeks Q-value = 0.07), and breastfeeding (R2: 1.6–2.6%, all Q-value ≤ 0.05, except 9 months Q-value = 0.1) had a continuous impact on microbiota composition throughout the first 11 months of life. Day care attendance emerged as a significant factor from 4 months of age (R2:1.4%, Q-value = 0.089), coinciding with the end of maternity leave in the Netherlands, and its effect persisted until the age of 14 months (R2: 2.0%, Q-value = 0.056). The presence of older siblings influenced the infant microbiota from the earliest time point (1–2 weeks of age, R2: 1.9%, Q-value = 0.026) up to 9 months of age (R2: 1.9%, Q-value = 0.028), which might suggest early household microbial transmission [59, 60]. In contrast, introduction of complementary feeding contributed to microbial variation only during the initial transition to complementary foods (4–6 months, R2: 1.5–1.8%, all Q-value < 0.1) but not thereafter, highlighting a transient effect at this developmental stage.

The pandemic began to influence infant microbial community composition at 5 months of age and became a statistically significant factor from 6 months onwards (PERMANOVA; Fig. 2B, Additional file 1: Table S12). The proportion of variance explained by the pandemic gradually increased over time, reaching a maximum of 2.7% at 11 months (Q-value = 0.006).

As the impact of the pandemic became more pronounced at later time points and persisted in sensitivity analysis performed using the samples collected at age 6 months (R2: 1.7%, Q = 0.046) (Additional file 2: Fig. S3, Additional file 1: Table S7), we further examined differences in bacterial species abundance between pre-pandemic and pandemic samples. The most pronounced shifts in species composition were observed at 9–14 months (Fig. 2C, Additional file 1: Table S13). Here, LinDa revealed that 44 out of 216 species were statistically significantly differentially abundant in samples collected during as compared to prior the start of the pandemic (Q-value < 0.2). The most pronounced depleted species in pandemic samples were *Actinomyces* sp*.* ICM47, particularly at 14 months (Q-value = 0.008, log2-fold change = −6.7), as well as 9 (Q-value = 0.004, log2-fold change = −6.6) and 11 months (Q-value = 0.017, log2-fold change = −6.3). Other taxa, including *Eggerthella lenta,* *Actinomyces odontolyticus* and *Gordonibacter pamelaeae*, also showed decreased relative abundances in pandemic samples at later time points.

Conversely, several species were significantly enriched in pandemic samples, notably *Bifidobacterium adolescentis* (9 months, Q-value = 0.028, log2-fold change = 7.8), *Ralstonia* sp*.* MD27 (9mon, Q-value = 0.002, log2-fold change = 7.0; 1wk, Q-value < 0.001, log2-fold change = 5.7), *Haemophilus parainfluenza* (9mon, Q-value = 0.017, log2-fold change = 6.1; 11mon, Q-value = 0.035, log2-fold change = 5.8) and *Streptococcus* sp A12 (11mon, Q-value = 0.016, log2-fold change = 5.6). These data further confirm a shift in microbial composition during the pandemic period.

### Linking behavioural exposure during the pandemic to shifts in infant gut microbial composition

To investigate whether shifts in bacterial species abundance during the pandemic were linked to changes in environmental exposure and parental behavior, we developed an EI based on questionnaire data from families with at least one infant fecal sample collected during the pandemic period (Additional file 2: Fig. S8). Longitudinal sample collection for each infant is summarized in Additional file 2: Fig. S9, showing individual sampling timelines relative to lockdown periods and the start of the COVID-19 pandemic in the Netherlands.

**Fig. 3:**
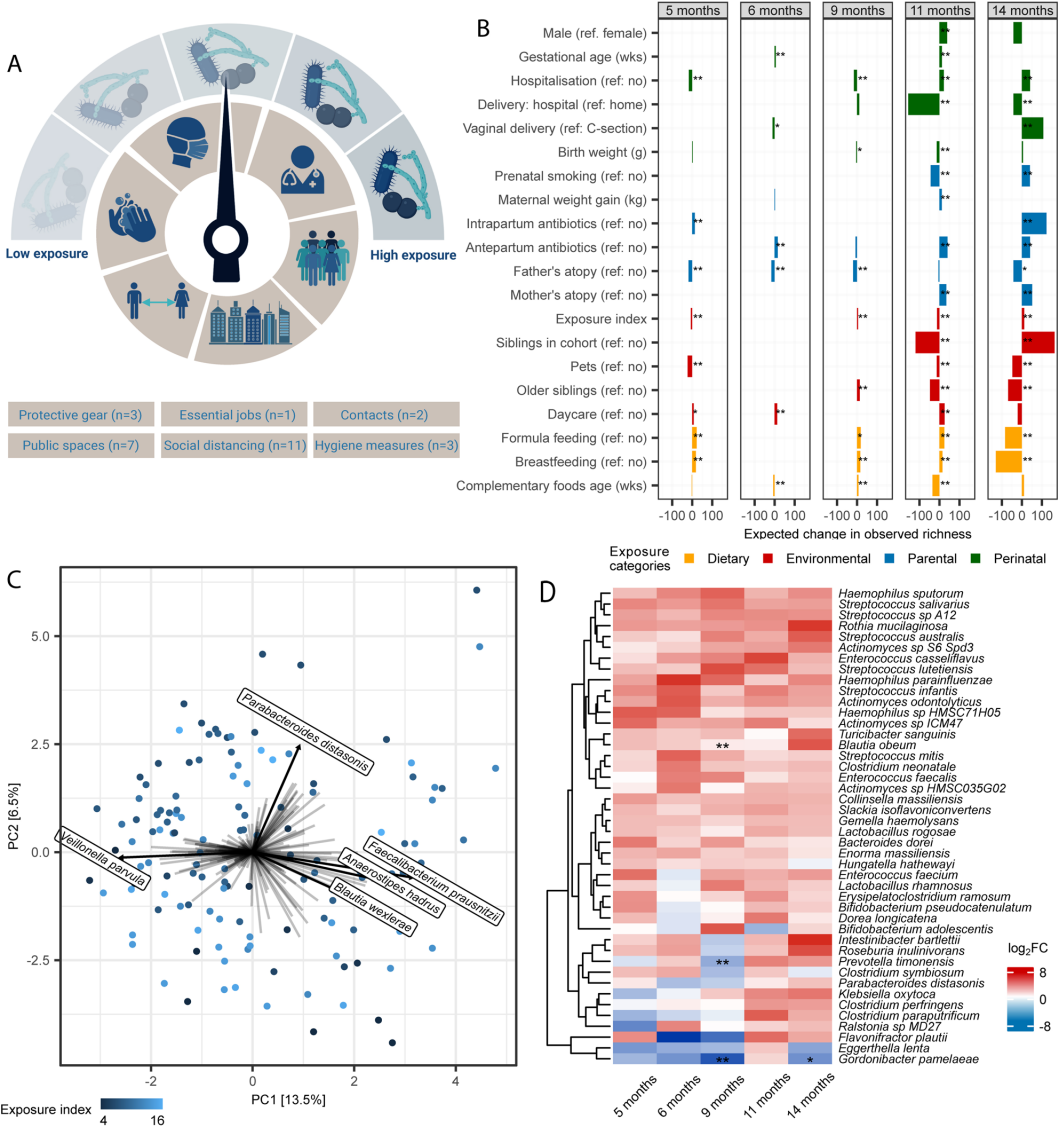
Pandemic Exposure index and its effect on the gut microbiota. **A** Graphical representation of the number and type of variables included in the EI. **B** Bar charts depicting regression coefficients of each variable in association to observed richness at 5–14 months for pandemic samples. Significant Q-values are indicated with stars: “**” indicates Q-values < 0.1, “**” indicates Q-values < 0.05. **C** Principal Component Analysis ordinating all 5–14-month fecal samples collected during the pandemic. The PCA was performed on centered log-ratio (clr)-transformed reads at the species level. Samples are color-coded by index values, with arrows representing taxon loading vectors driving the largest variation along the first two Principal Components.** D** Heat-map visualizing how the 44 bacterial species which were differentially abundant in pandemic samples at 5–14 months of age are associated with the EI as continuous predictor (LinDa). The color gradient ranges from blue (lower abundance) to red (higher abundance), representing log changes. Statistically significantly differentially abundant species are indicated with stars: “**” indicates Q-values < 0.05 and “*” for Q-values between 0.05 and 0.2. Log_2_ FC: Log_2_ Fold Change

The final EI (Additional file 2: Fig. S10), derived from 36 families, consisted of 10 grouped variables (Fig. 3A) and followed a normal distribution (Shapiro–Wilk test: *p*-value = 0.171, W = 0.957). Scores ranged from 4 to 18 (mean = 11.4, SD = 3.8), with higher values reflecting greater exposure—characterized by fewer hygiene measures, reduced protective behaviors, and increased social interactions (Additional file 1: Table S4).

With increasing EI—indicating greater environmental exposure—we observed significant shifts in microbial richness across multiple time points. Upon adjustment for other covariates, richness decreased at 5 months (estimate = −6.9, 95% CI [−11.4, −2.5], Q-value = 0.015) and 11 months (estimate = −11.9, 95% CI [−16.2, −7.6], Q-value = 0.004), whereas an increase in microbial richness was detected at 9 months (estimate = 4.8, 95% CI [0.9, 8.7], Q-value = 0.031) and 14 months (estimate = 11.9, 95% CI [2.8, 20.9], Q-value = 0.033) (Fig. 3B, Additional file 1: Table S14).

Similarly, effective number of species (ENS) decreased at 6 months (estimate = −1.5, 95% CI [−2.5, −0.4], Q-value = 0.027), 9 months (estimate = −3.9, 95% CI [−4.9, −3.0], Q-value = 0.002), and 11 months (estimate = −1.6, 95% CI [−2.9, −0.2], Q-value = 0.047), while increasing at 14 months (estimate = 3.6, 95% CI [0.9, 6.2], Q-value = 0.035) (Additional file 2: Fig. S11, Additional file 1: Table S14).

We analysed beta-diversity in pandemic-era samples collected between 5 and 14 months of age, the period when the pandemic effect was most pronounced. Although no clear overall separation by EI was observed, likely due to the dominant influence of age on sample clustering in PCA space (Fig. 3C), at 6 months the EI accounted for 6.8% of the variation (Q-value = 0.019, Additional file 2: Fig. S12, Additional file 1: Table S15).

Next, we assessed the impact of EI on the abundance of bacterial species that were significantly differentially abundant in pandemic-era samples. Of the 44 species identified, *Blautia obeum* and *Prevotella timonensis* were excluded as too few samples contained these species. Using EI as a continuous predictor, we observed that higher EI was associated with reduction in *G. pamelaeae* abundance at 9 months (Q-value = 0.024; fold change = − 4.7) and 14 months (Q-value = 0.16; fold change = − 6.3) (Fig. 3D; Additional file 1: Table S16, Additional file 2: Fig. S13). A subsequent comparison was made using EI dichotomized at the mean (> 11.48 vs ≤ 11.48) as a visualization of the same association and to place it in the context of pre-pandemic samples (Additional file 2: Fig. S14). In this broader comparison, *G. pamelaeae* abundance is highest in pre-pandemic samples and in pandemic-era infants with low EI, consistent with a U-shaped pattern across the three groups: prepandemic, above index and below index mean.

Finally, we assessed whether the pandemic-associated differences in the infant gut microbiota varied by (i) the presence of older siblings in the household and (ii) the cumulative duration of pandemic exposure up to faecal sampling. Stratifying by older siblings attenuated many associations, consistent with reduced statistical power. However, we observed no evidence of stronger pandemic-related effects among infants without older siblings as compared to infants with siblings: neither the proportion of variance explained in community composition (Additional file 1: Table S17) nor the estimated differences in microbial richness (Additional file 1: Table S18) were larger in this group.

Among samples collected during the pandemic, we evaluated cumulative exposure to pandemic conditions by modelling the time elapsed (in days) between the start of the pandemic and the sample collection date as a dichotomized explanatory variable. In these within-pandemic analyses, cumulative exposure was significantly associated with overall microbial community structure at 6 months (PERMANOVA R^2^ = 6.9%, Q-value = 0.004, Additional file 1: Table S19, Additional file 2: Fig. S15), with similar but non-significant trends at 4 and 14 months. Furthermore, longer time since the start of the pandemic was associated with lower microbial richness at multiple ages, reaching statistical significance at 1–2 weeks, 4 weeks, 4, 5, and 14 months (Q-value < 0.1; Additional file 1: Table S20, Additional file 2: Fig. S16). Overall, these sensitivity analyses support the hypothesis that duration of exposure to pandemic conditions may contribute to microbiome variation, with longer time in the pandemic associated with lower richness across multiple ages and a significant association with community structure at 6 months.

## Discussion

In this study, we made use of the unique timing of the COVID-19 pandemic to explore how abrupt lifestyle, and behavioural changes affected the gut microbiome development during infancy. Within the well-phenotyped prospective LucKi Gut study, we showed that microbiota maturation followed age-related trajectories that aligned with previous studies [8, 13] but was measurably impacted by the pandemic from 6 months of age onwards.

First, we investigated infant gut microbiota development. As anticipated, microbial richness and diversity increased with age [54–57], and community composition matured toward a more complex, adult-like profile over time. Our results align with previous studies demonstrating distinct microbial phases in infancy, characterized by early dominance of *Bifidobacterium* species [61] and a gradual increase of other taxa including *Bacteroides*, *Faecalibacterium*, and *Collinsella* upon cessation of breastfeeding and the introduction of complementary foods [54–57].

We, moreover, confirmed well-established associations between birth mode [55, 62], infant feeding [8, 10], daycare attendance [63] and older siblings [64] with infant microbiota composition, further validating the robustness of our data.

Second, we investigated the effect of the pandemic on infant gut microbiota maturation. To our knowledge, this is among the first large-scale longitudinal study using whole metagenome sequencing to assess the pandemic’s impact on infant gut microbiota development.

Notably, microbial richness was significantly increased in pandemic samples at 9 months of age, and community composition began to diverge measurably from pre-pandemic samples at 6 months. Moreover, 44 bacterial species differed significantly in abundance between pre- and post-pandemic fecal samples of infants of the same age. Together this suggests that reduced exposure to social and environmental microbes during the pandemic may have disrupted typical microbial succession. This effect becomes mainly apparent once infants are weaned and the dominant effect of infant feeding on the microbiome composition starts to diminish. Although infants raised during the COVID-19 pandemic were less frequently exposed to antibiotics at 9 and 11 months of age, this is unlikely to explain the observed associations between the pandemic and infant gut microbiota composition, as antibiotic exposure was included as confounder in our models. Instead, the reduced antibiotic use further exemplifies the reductions in pathogen transmission during the pandemic.

Interestingly, several *Actinomyces* species were significantly lower in pandemic as compared to pre-pandemic samples at later time points. Bailey et al. previously reported a positive correlation between fecal *Actinomyces* levels and air pollution levels, particularly with particulate matter PM_2.5_ and gas NO_2_ [65]. The significant global reduction in air pollution during the pandemic, including reductions in PM_2.5_ and NO_2_ emissions [66, 67], could potentially explain the decreased levels of *Actinomyces* in pandemic samples. Beyond reducing environmental pollution, the pandemic has resulted in various changes in social interactions and hygiene practices that might have all affected infant gut microbiota. To explore how variation in family behavior during the pandemic contributed to microbiota outcomes, we next developed a novel EI capturing hygiene practices, social contacts, and lifestyle changes.

Interestingly, higher EI scores—indicating more environmental exposure and fewer protective measures—were associated with both increased and decreased microbial richness, depending on the infant's age. While these associations might seem inconsistent, they likely reflect age-dependent windows of microbial vulnerability or resilience.

We speculated that pandemic effect may be cumulative, depending on the duration of exposure to pandemic-related conditions such as social distancing and lifestyle changes and that this difference in cumulative exposure could contribute to the inconsistencies we observed in alpha diversity metrics, where the impact differed across ages. Consistent with this, our within-pandemic analyses showed that longer time since the start of the pandemic was consistently associated with lower microbial richness across multiple ages and with differences in community structure at 6 months, suggesting an exposure–response pattern operating within the pandemic period.

*G. pamelaeae* was overall less abundant in infants sampled during the pandemic compared with pre-pandemic infants, indicating a pandemic-era downward shift in baseline levels. Within the pandemic cohort, a higher EI was nonetheless associated with lower *G. pamelaeae* abundance, suggesting that EI-related variation operates on top of this shifted baseline. Thus, the overall pandemic-era depletion of this species and its inverse association with EI within the pandemic likely reflect distinct, potentially non-linear ecological processes whereby higher EI may increase microbial turnover and competition (e.g., priority effects or ecological displacement) and correlate with lifestyle routines that shape its niche rather than a simple monotonic exposure–abundance relationship.

Given its role in bile acid metabolism and production of immunomodulatory compounds such as 3-oxolithocholic acid and urolithins [68–70] reduced abundance of *G. pamelaeae* could have functional implications for immune development. While speculative, our findings raise the possibility that altered microbial exposure may impact immune programming via loss of specific microbial functions. To uncover such functional consequences, future studies are needed that include fecal metabolomics and immune profiling.

Prior to our study, several cross-sectional studies and cross-cohort comparisons have explored the association between the pandemic and the infant gut microbiota. A study comparing a socioeconomically and racially diverse cohort of 54 American 12-month-old infants [30] reported a lower abundance of *Haemophilus* in fecal samples collected during the pandemic. The authors attributed this decrease to more intensive cleaning and disinfection practices. In contrast, our results showed higher abundance of several *Haemophilu*s species in pandemic samples. Specifically, we observed significant increases in *H. parainfluenzae*, and *H. sputorum* at 9 months and *Haemophilus* sp. HMSC71H05 at 11 months of age. Our findings are consistent with another study [63] showing that infants cared for at home have higher levels of *Haemophilus* than those attending daycare. This pattern may help explain the discrepancy between our results and the American cohort and underscores the need for caution in generalizing results across cohorts.

The Irish CORAL study reported lower levels of environmentally transmitted clostridia, including *Hungatella*, and higher levels of fecal bifidobacteria in their pandemic infant cohort as compared to pre-pandemic cohorts [31]. We also observed reduced levels of *Hungatella hathewayi* in 11-month-old infants during the pandemic, while levels of *Bifidobacterium pseudocatenulatum* and *B. adolescentis* were enriched between 9 to 14 months of age among infants sampled during the pandemic.

In parallel, a small number of longitudinal studies in adults suggest that pandemic restrictions can also measurably shift the gut microbiota, plausibly via altered social/environmental exposures, stress and lifestyle changes. In healthy adults assessed pre- vs post-lockdown, increased anxiety and stress coincided with reduced gut alpha diversity and increased Proteobacteria, suggesting a stress-related perturbation of the gut microbiota [71]. Similarly, in Brazilian older women followed across 6 months of physical distancing, targeted profiling showed a selective increase in *Blautia* spp., alongside measurable lifestyle and immune-marker changes [72].

Finally, in a within-person metagenomic study spanning pre-pandemic travel and the first pandemic wave, participants showed shifts in taxonomic richness (including lower Actinomycetota richness) and changes in taxonomic and resistome profiles during the pandemic period [73]. Together with the infant cross-cohort findings [30–32], these adult data support the broader notion that abrupt, population-wide changes in contact patterns and hygiene can influence gut community structure.

Altogether, our findings are partly consistent with, yet extend, the few previous longitudinal adult and cross-sectional/cross-cohort infant studies [30–32] by incorporating dense longitudinal data and whole metagenome shotgun sequencing. Unlike these previous studies, we could demonstrate that pandemic effects emerge gradually and are most pronounced during later stages of infant microbial development. This refined understanding was facilitated by some of the key strengths of our study, including its longitudinal design, the application of deep sequencing, and the use of a detailed behavioral exposure index. However, some limitations should be acknowledged. The exposure index was available for a subset of 36 families, potentially limiting power. Moreover, this index has not been validated against objective measures of microbial exposure and, while it likely captures meaningful variation across participants, it may not fully reflect true exposure levels.

In addition, we lacked concurrent functional (e.g., metabolomics) and immunological data to determine the physiological consequences of compositional shifts, particularly regarding the potential roles of *G. pamelaeae*.

## Conclusions

In conclusion, by drastically altering patterns of social and environmental exposure, the COVID-19 pandemic provided a natural experiment to explore determinants of early-life microbiome development. Our findings indicate that such abrupt shifts in exposure can profoundly affect the infant gut microbiome during critical windows of microbiome maturation. Future research should examine whether these compositional changes translate into functional or clinical consequences later in life.

## Supplementary Information

Additional file 1: Supplementary Tables S1-S20.

Additional file 2: Supplementary Figures S1-S16.

## Data Availability

Trimmed and quality filtered reads, with removed human reads can be found at European Nucleotide Archive under project number PRJEB89491 [74]: https://www.ebi.ac.uk/ena/browser/view/PRJEB89491 Code availability: To support reproducibility, all code used for data processing, analysis, and figure generation is available in the project GitHub repository [75]: https://github.com/MUMC-MEDMIC/LuckiGut/tree/main/Covid_paper_scipts The authors declare no competing interests.Participant-level metadata are not publicly available due to privacy and re-identification risks and are available only under controlled access. Qualified researchers may request access through an independent data access committee (email to: luc.smits@maastrichtuniversity.nl), which will evaluate requests for valid research purposes, subject to review and execution of a Data Transfer Agreement between Maastricht University and the requester’s institution, with an estimated turnaround time of 2 months. The DTA includes restrictions on re-identification, onward sharing, and use outside the approved project.

## Abbreviations

BH: Benjamini-Hochberg
CLR: Center Log Ratio
DDS: Dietary Diversity Score
DMM: Dirichlet Multinomial Mixture
EI: Exposure Index
ENS: Effective Number of Species
FDR: False Discovery Rate
FVR: Food Variety Score
IQR: InterQuartile Range
LinDA: Linear models for Differential Abundance analysis
NMDS: Non-metric MultiDimensional Scaling
PC: Principal Components
PCA: Principal Component Analysis
PERMANOVA: PERmutational Multivariate ANalysis Of VAriance
