# Supplementary material for: The impact of the COVID-19 pandemic and associated lifestyle changes on early-life microbiome development
Source: Genome Med. 2026 Apr 29;18:86. doi: 10.1186/s13073-026-01660-8 (PMC13267274; doi:10.1186/s13073-026-01660-8)

## Slide 1
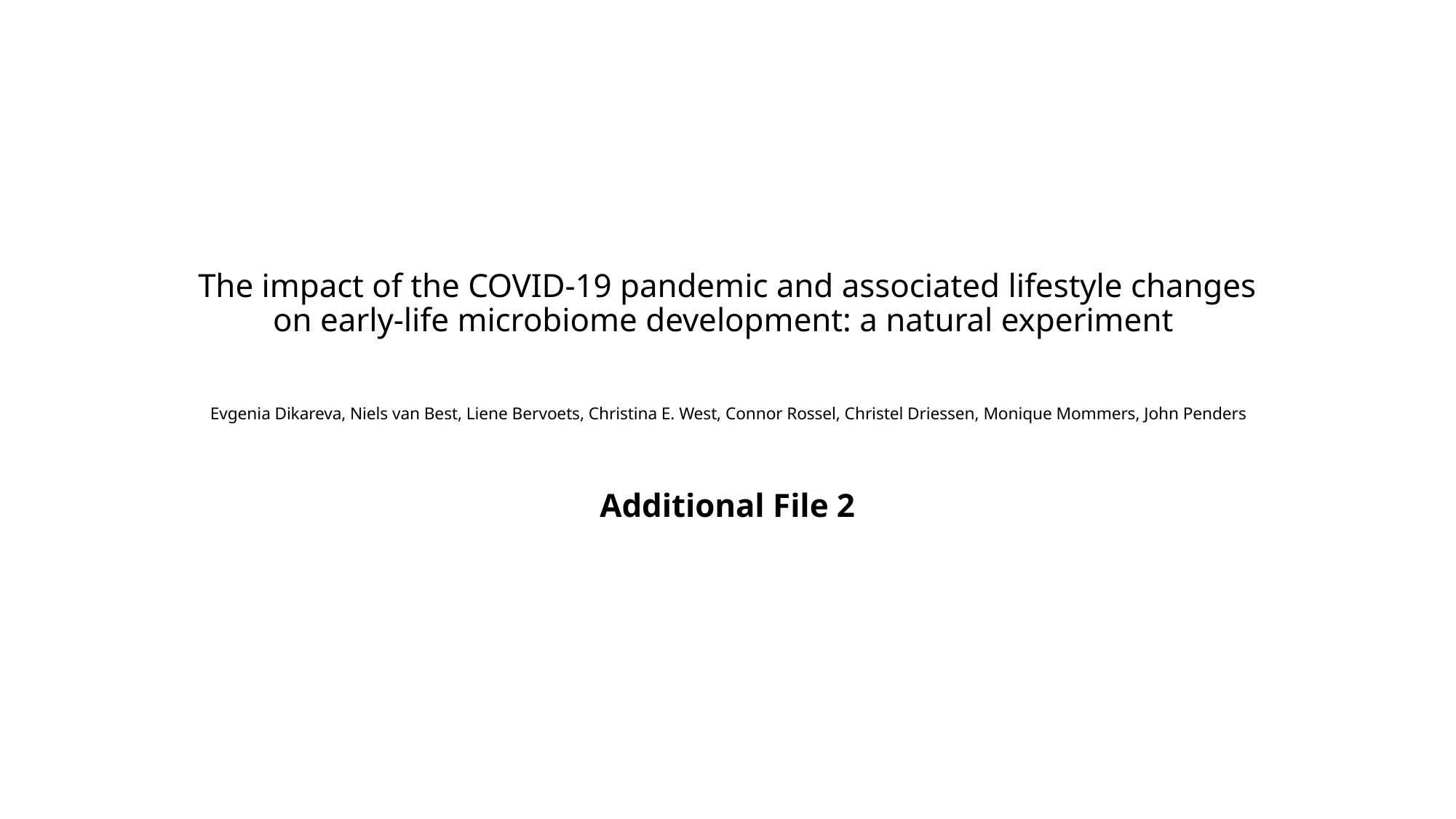

# The impact of the COVID-19 pandemic and associated lifestyle changes on early-life microbiome development: a natural experiment   Evgenia Dikareva, Niels van Best, Liene Bervoets, Christina E. West, Connor Rossel, Christel Driessen, Monique Mommers, John Penders Additional File 2

## Slide 2
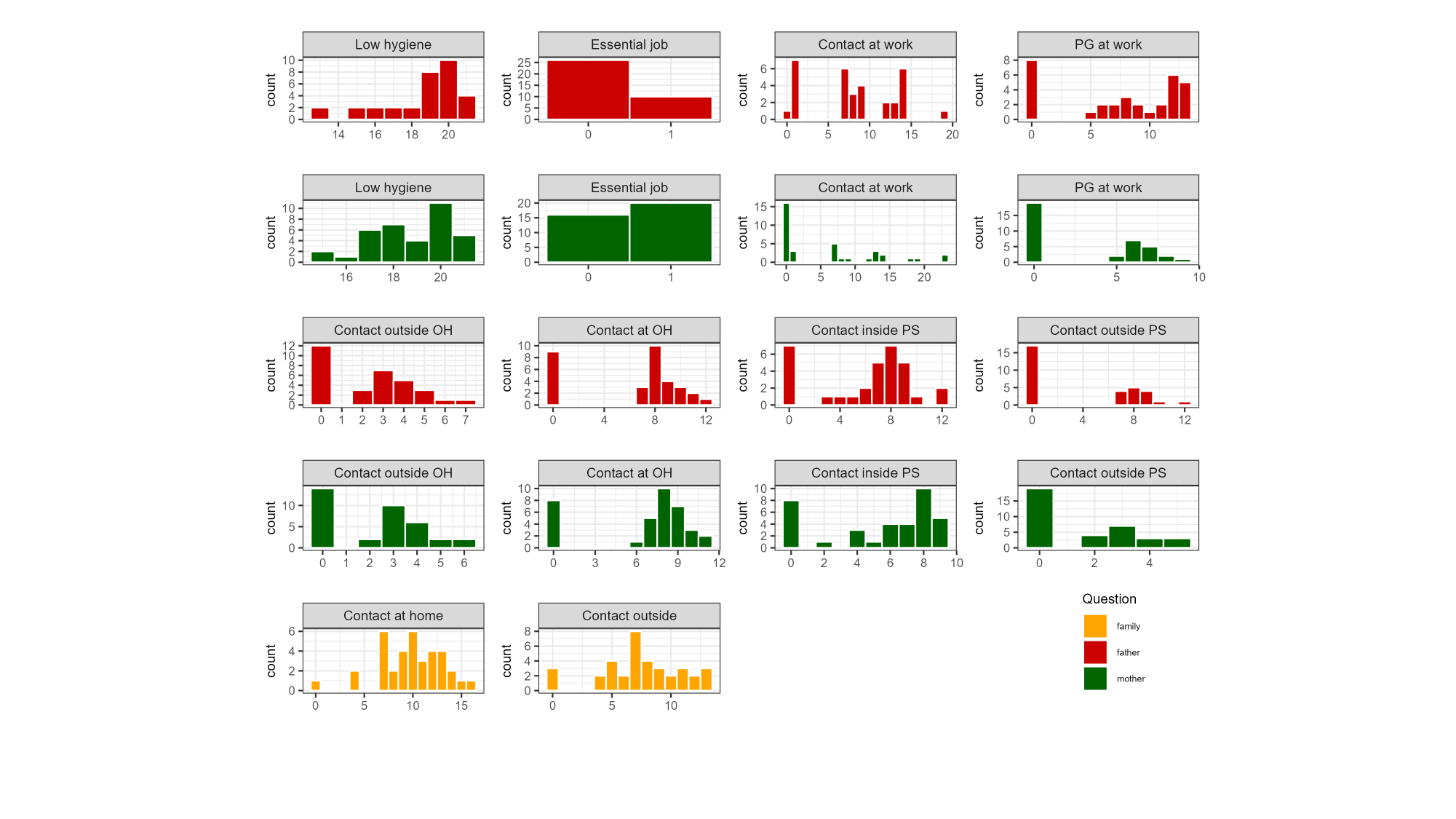

## Slide 3
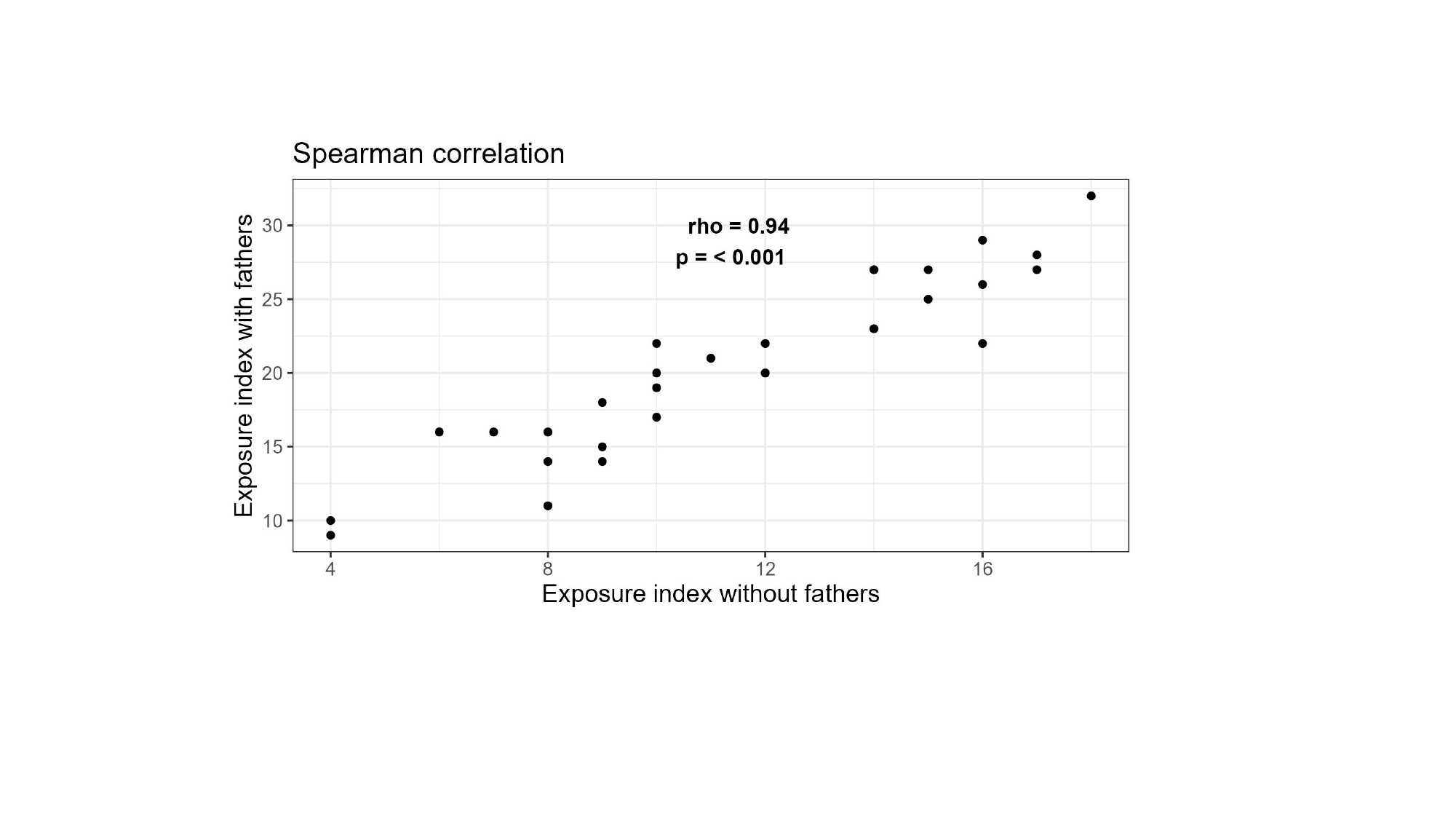

## Slide 4
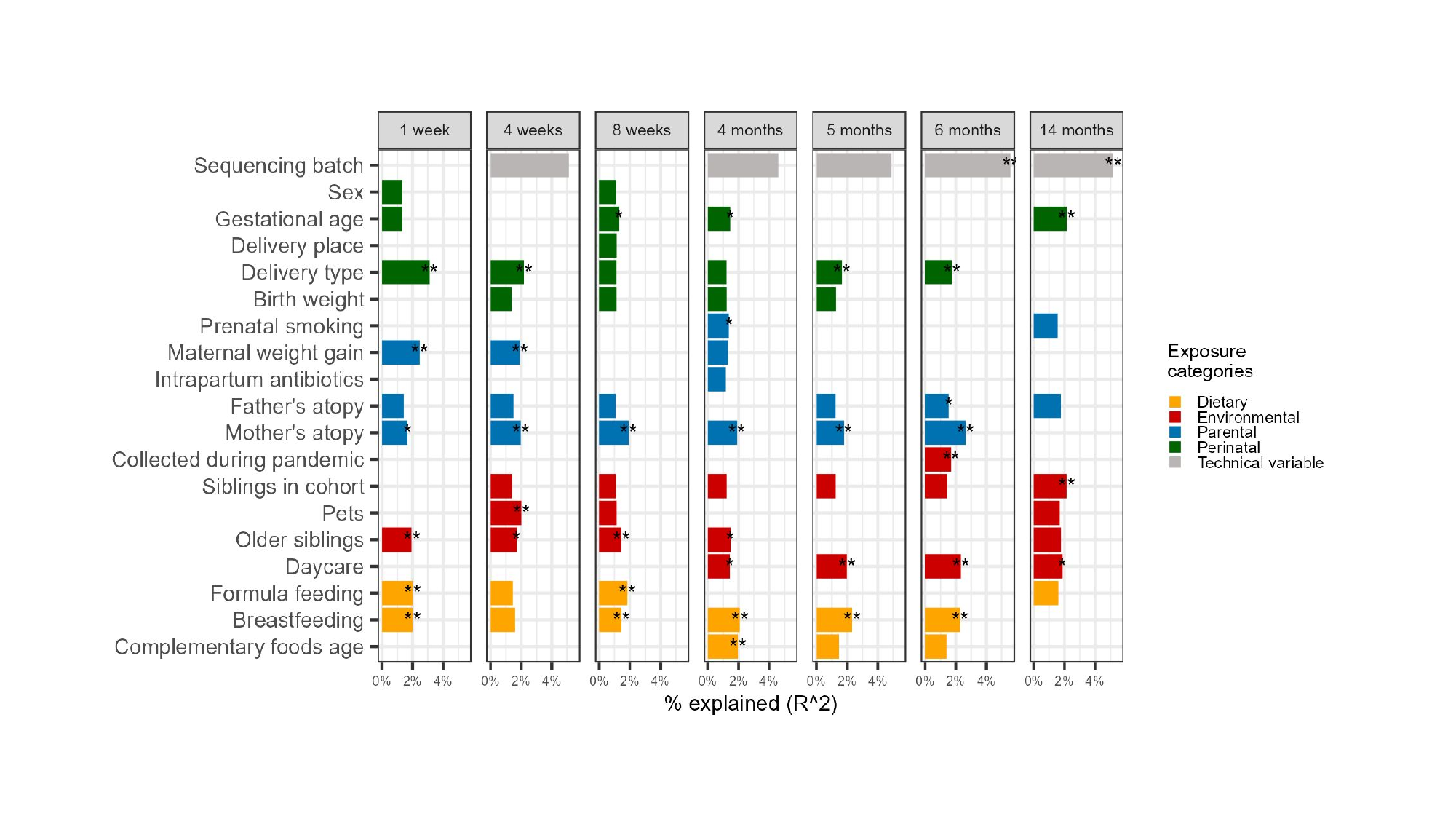

## Slide 5
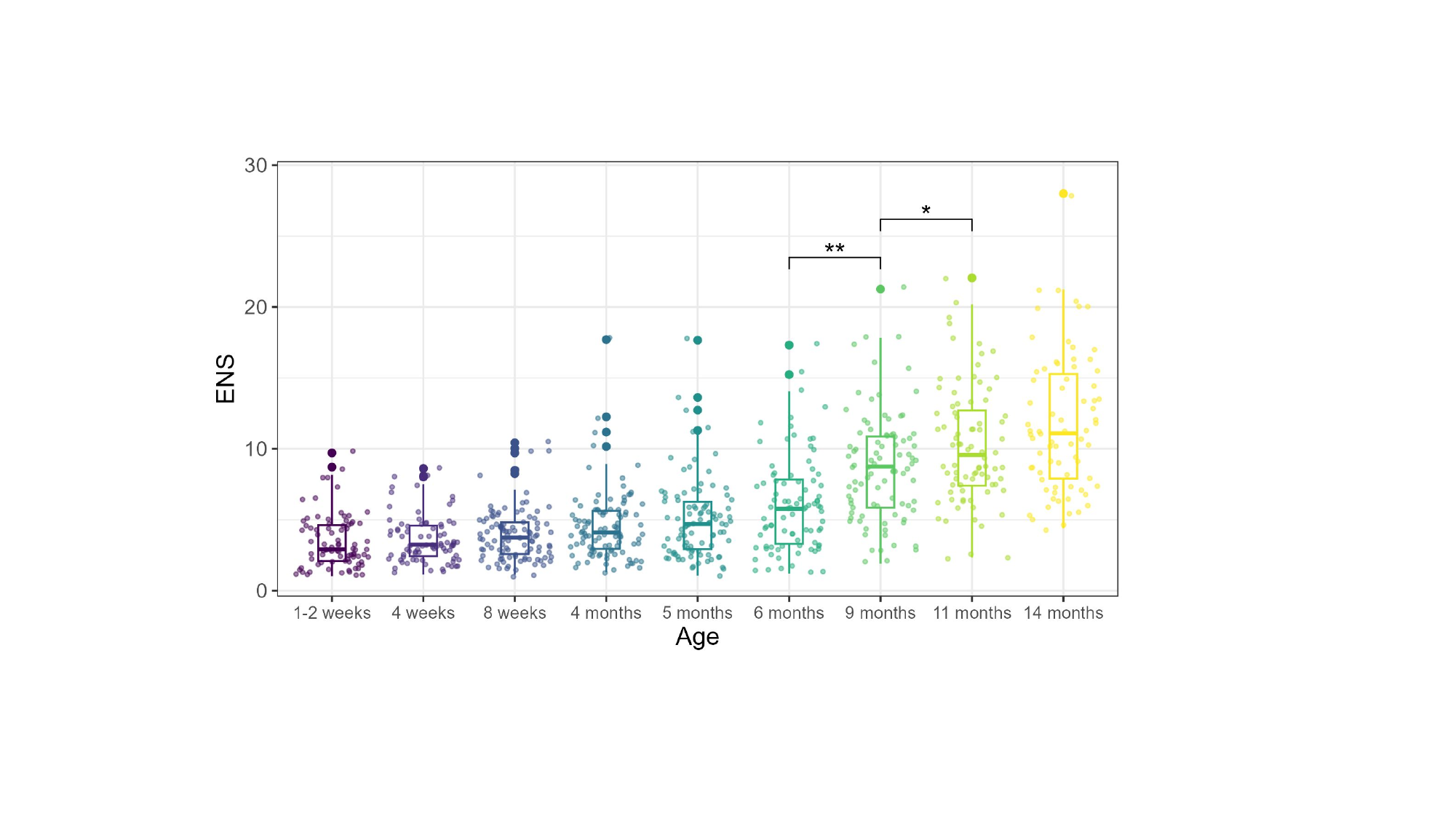

## Slide 6
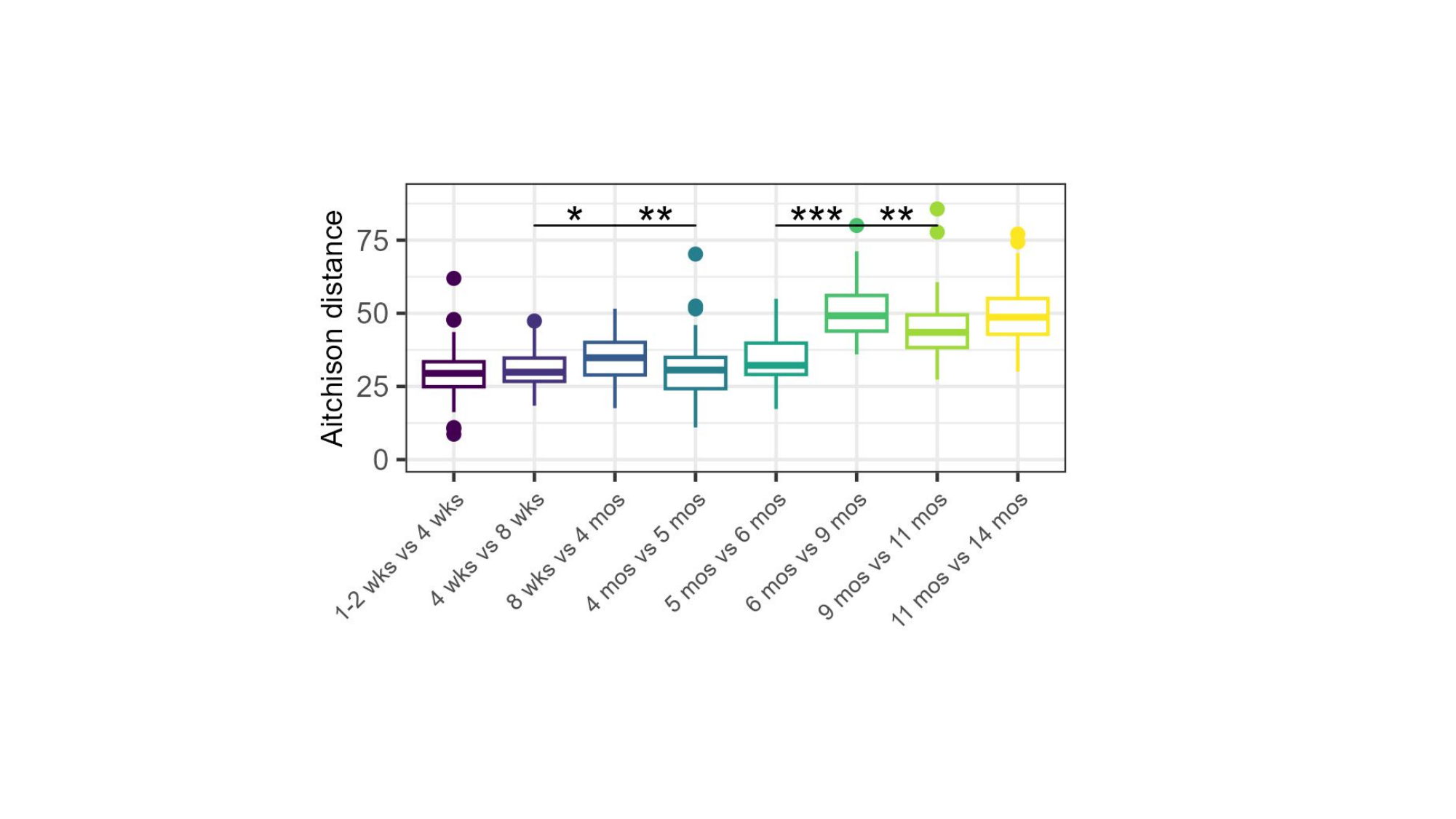

## Slide 7
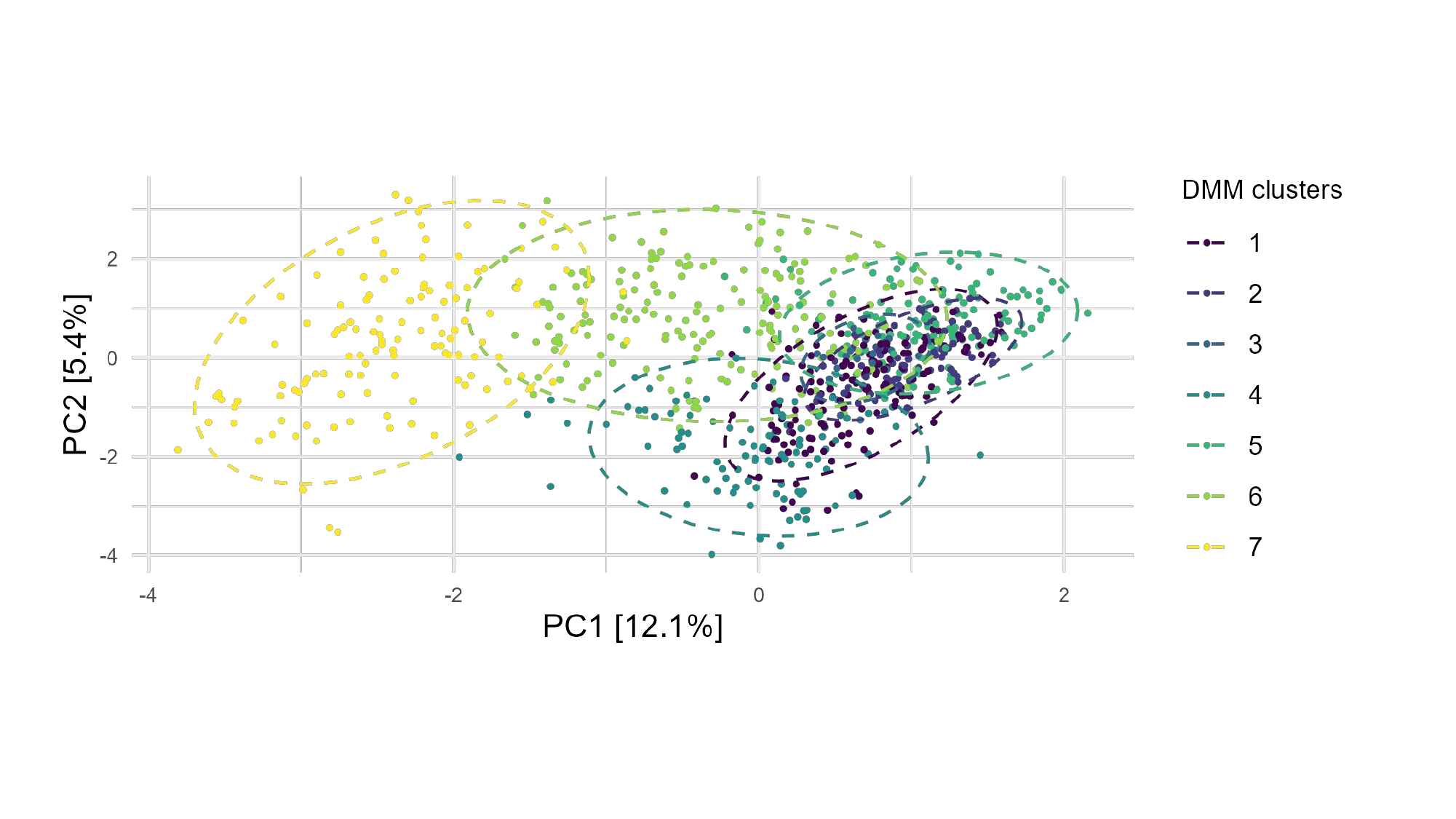

## Slide 8
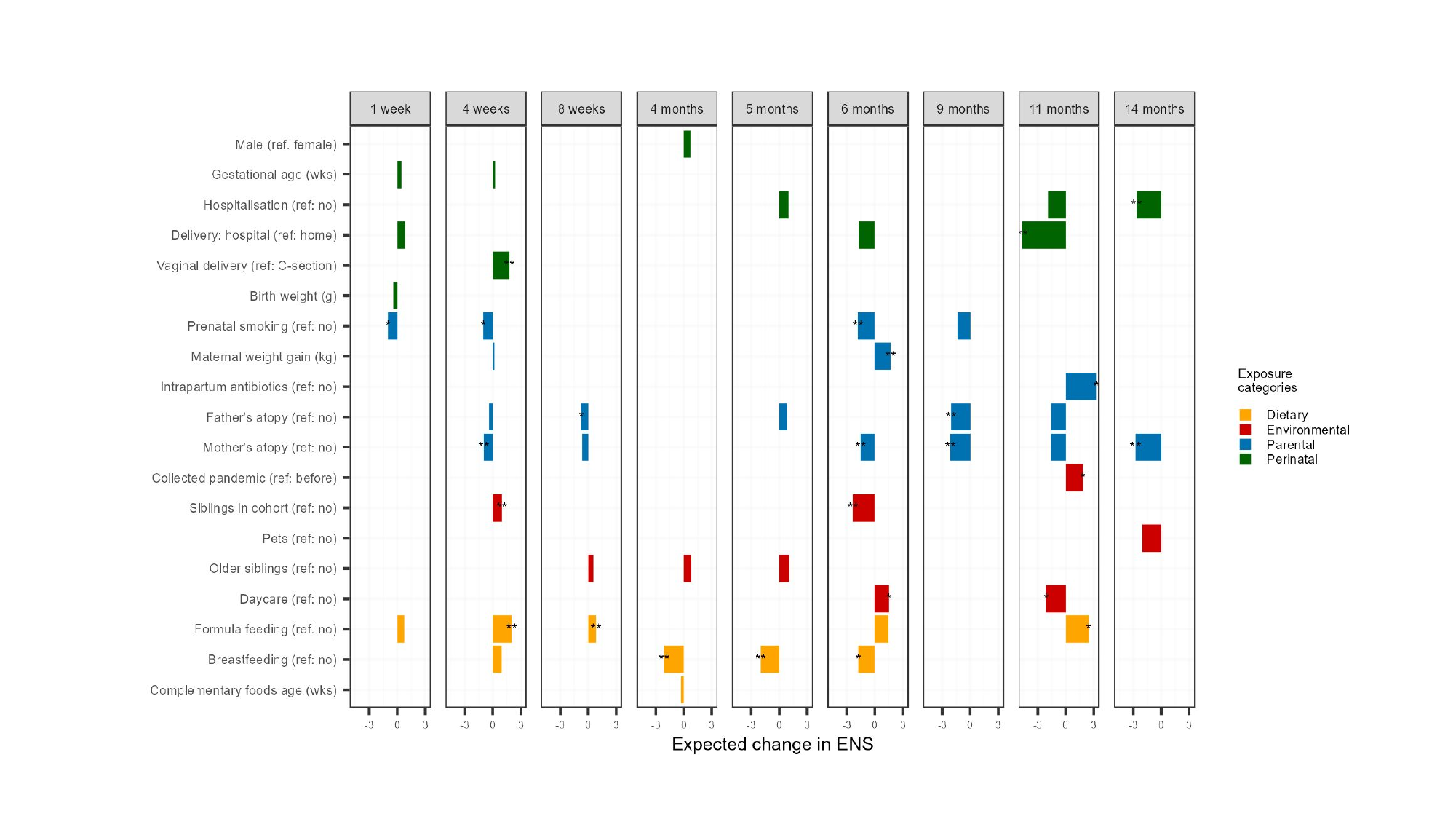

## Slide 9
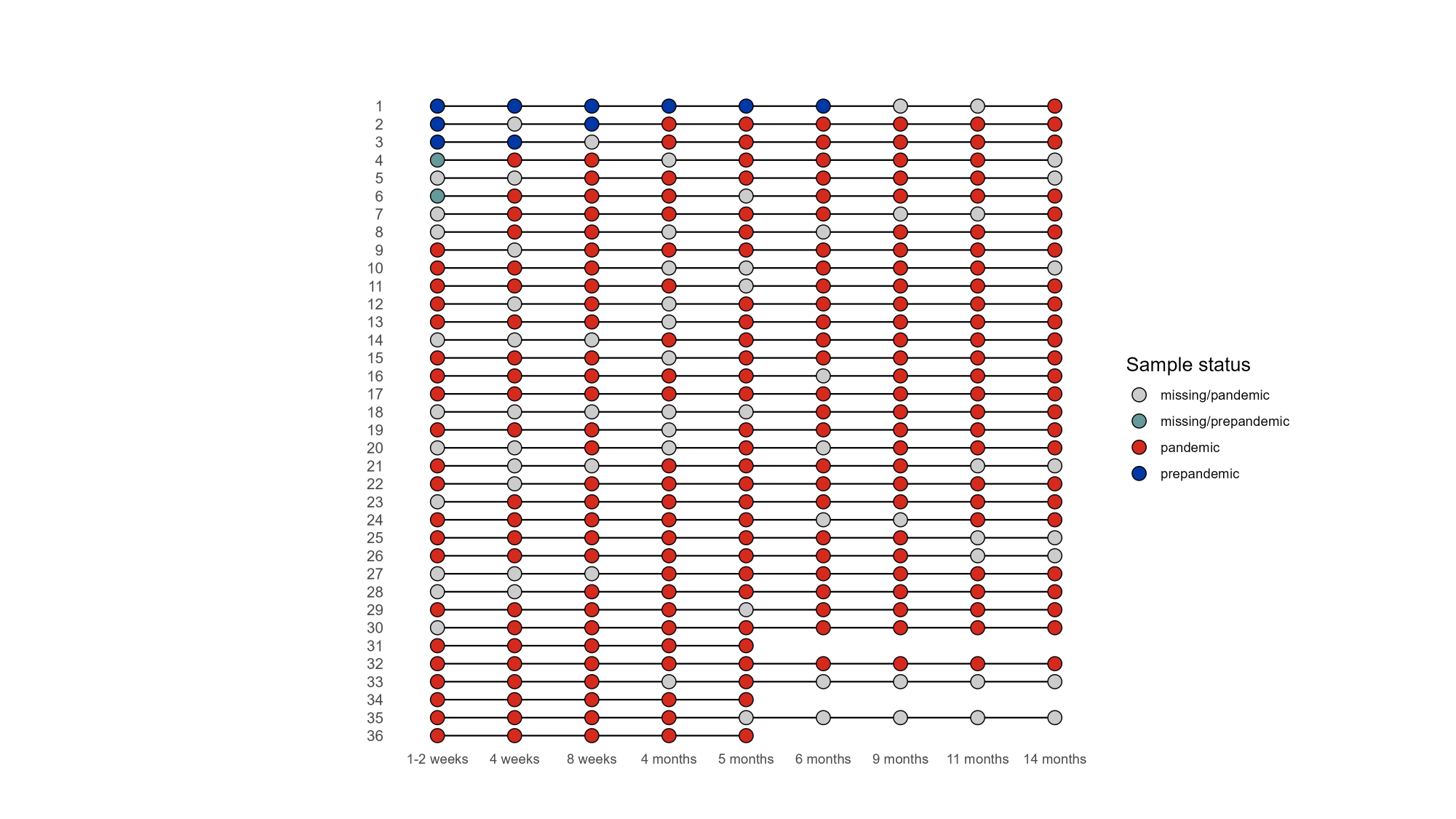

## Slide 10
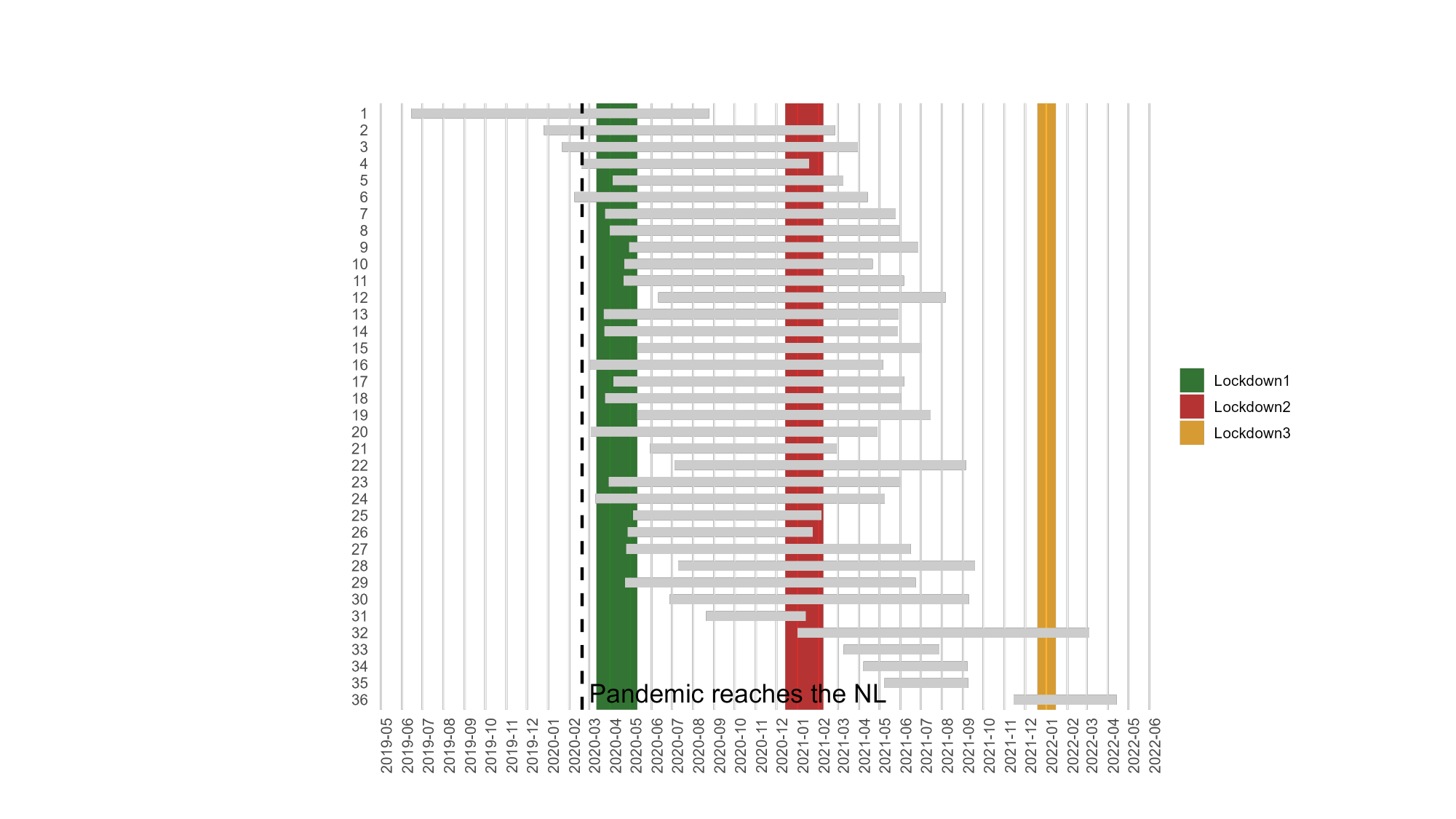

## Slide 11
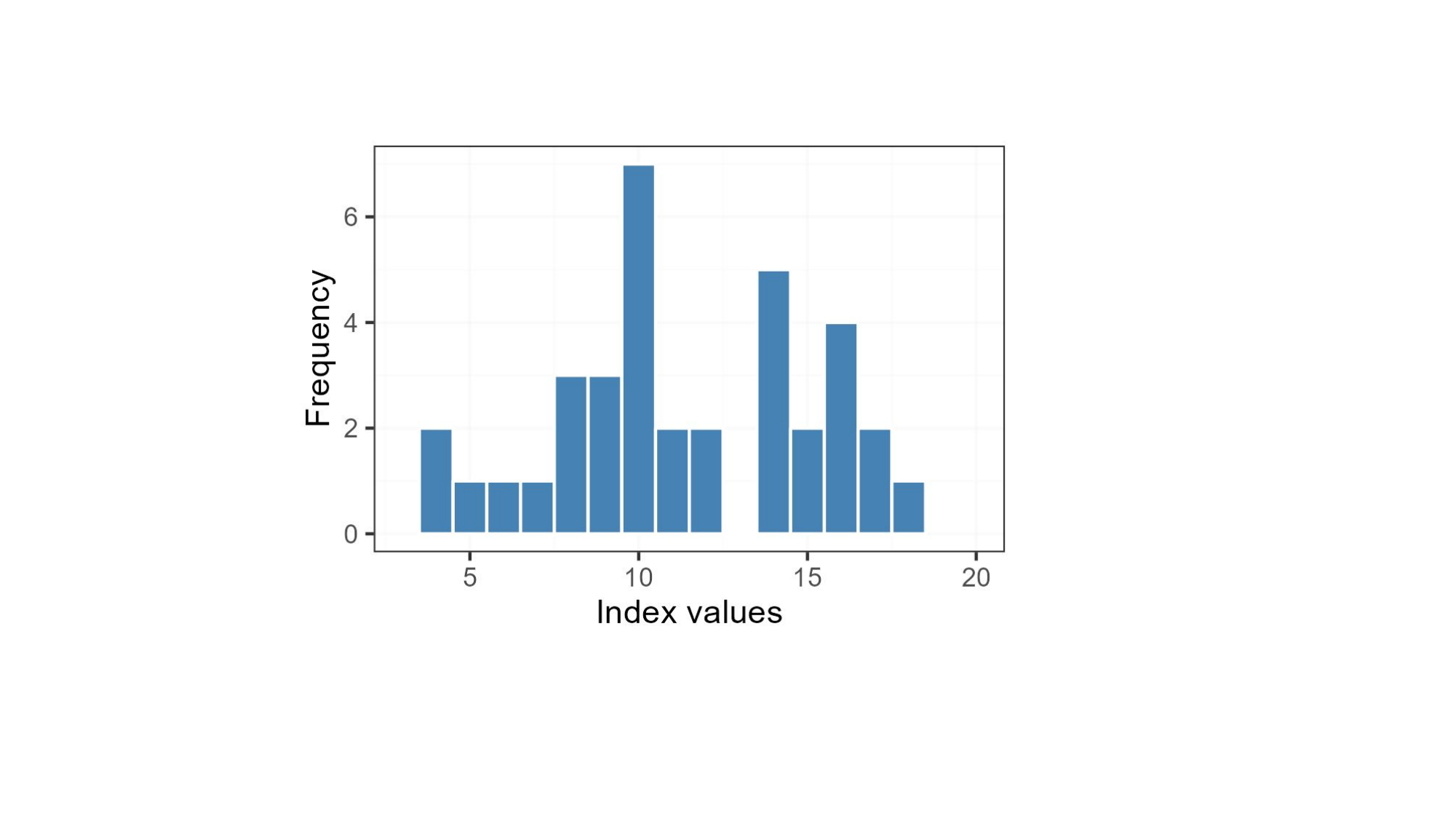

## Slide 12
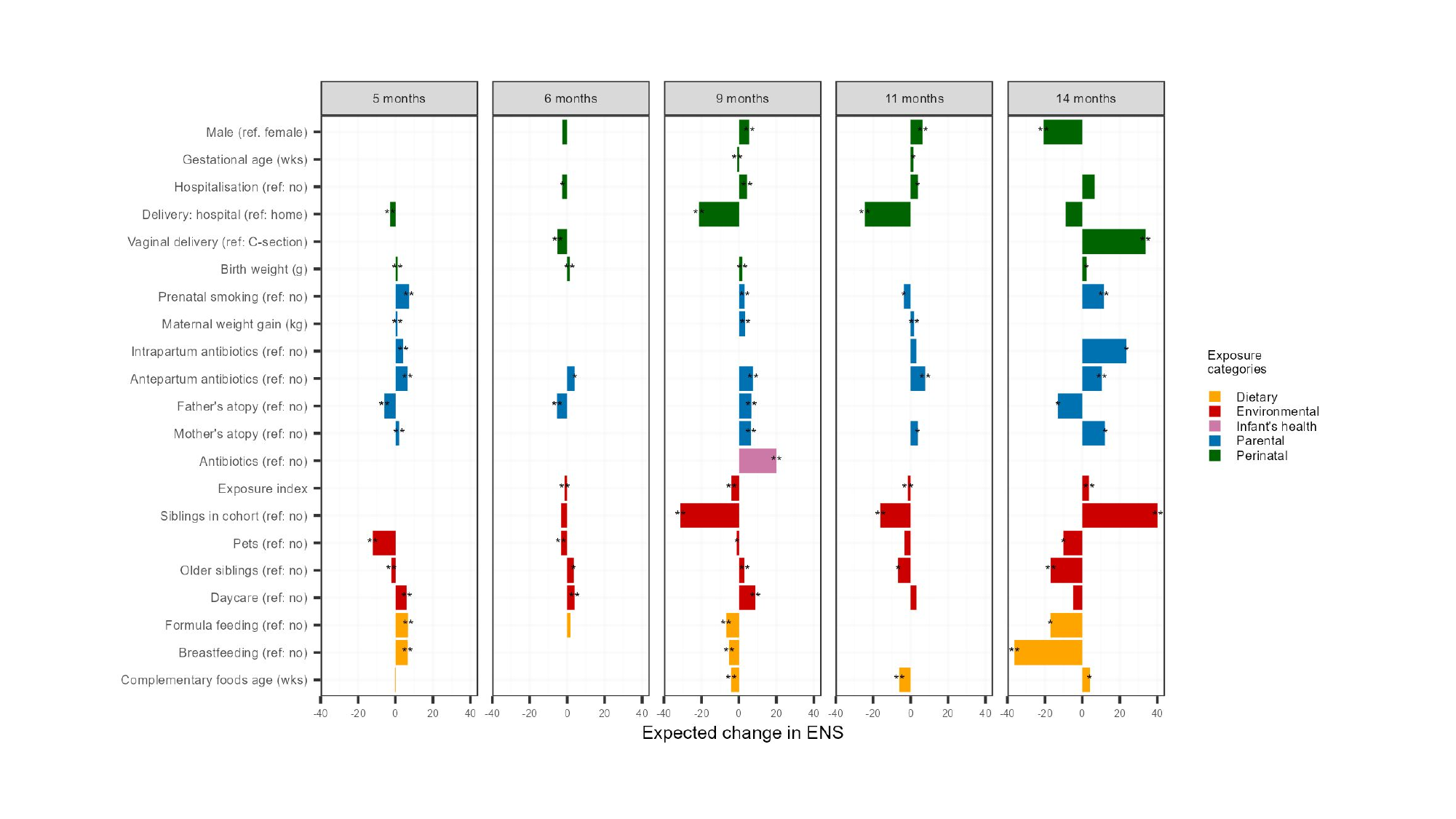

## Slide 13
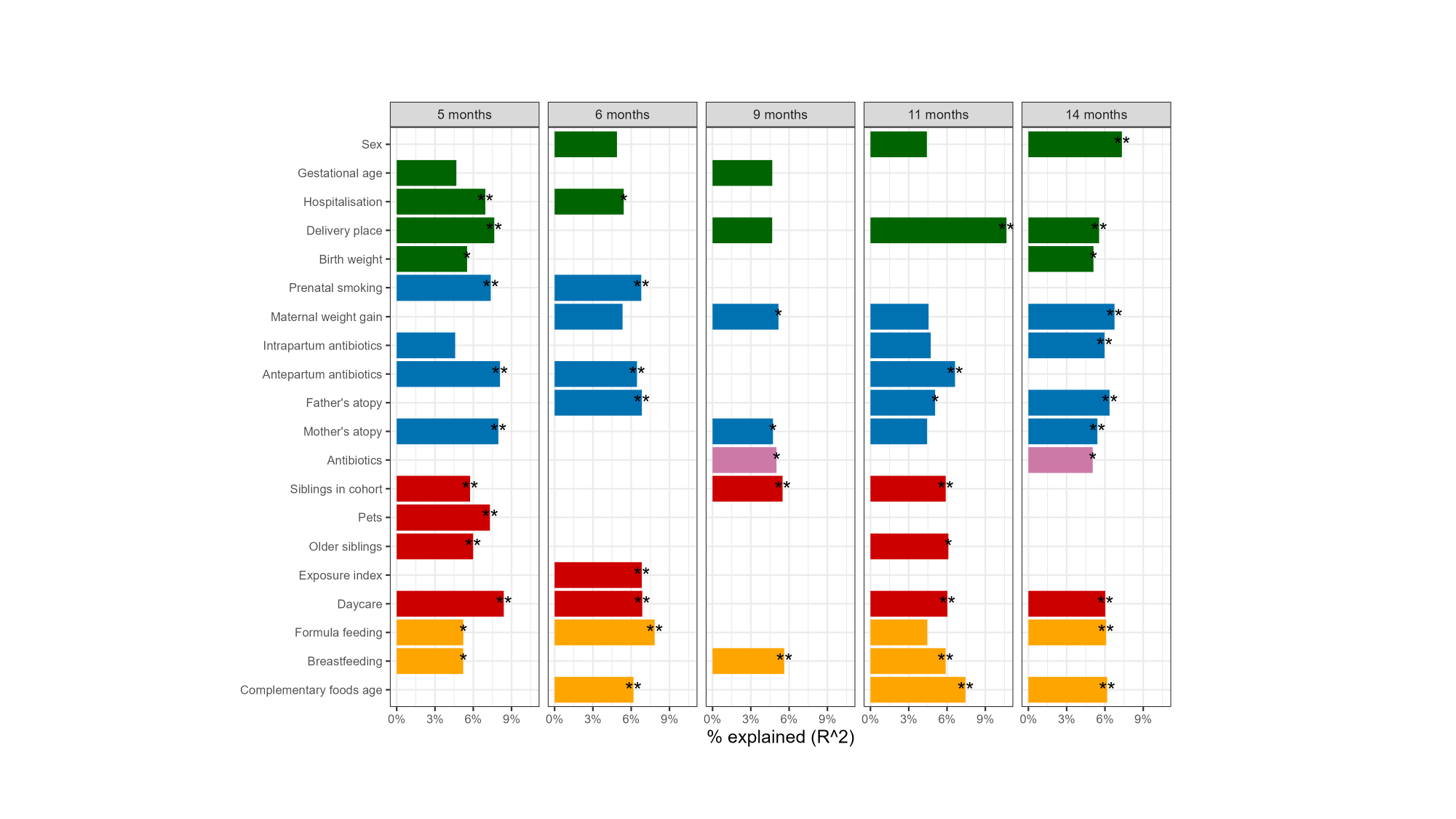

## Slide 14
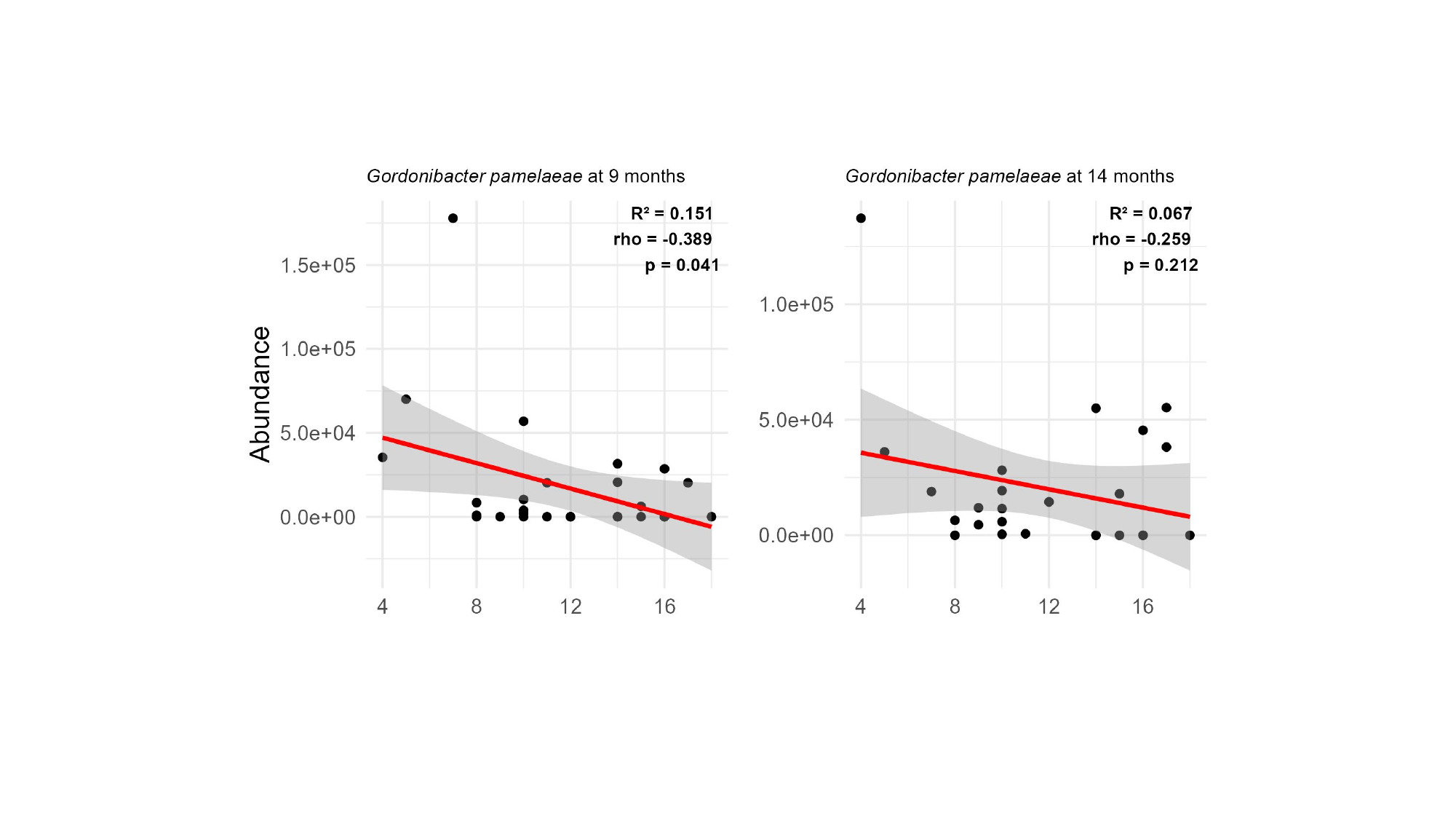

## Slide 15
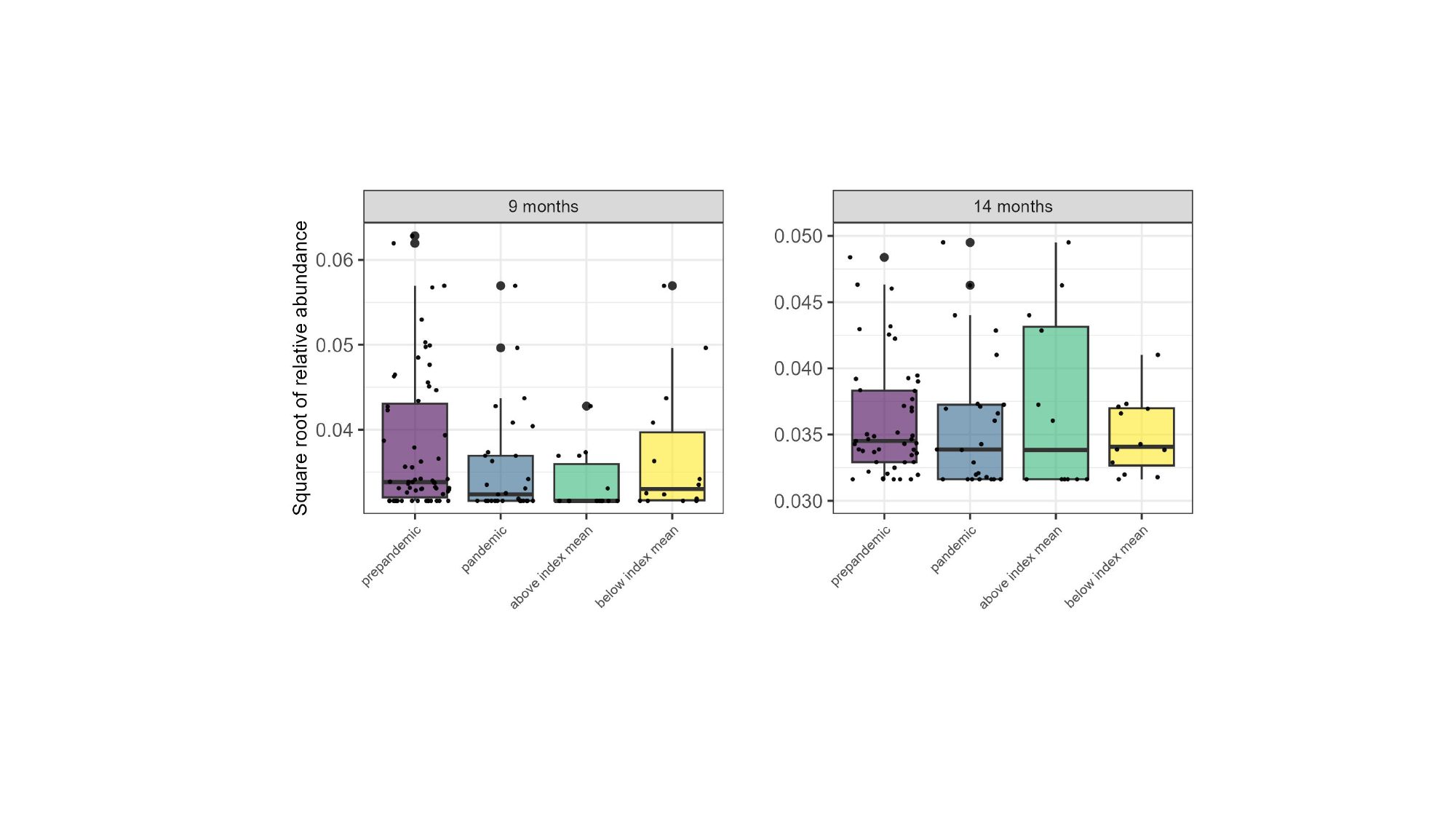

## Slide 16
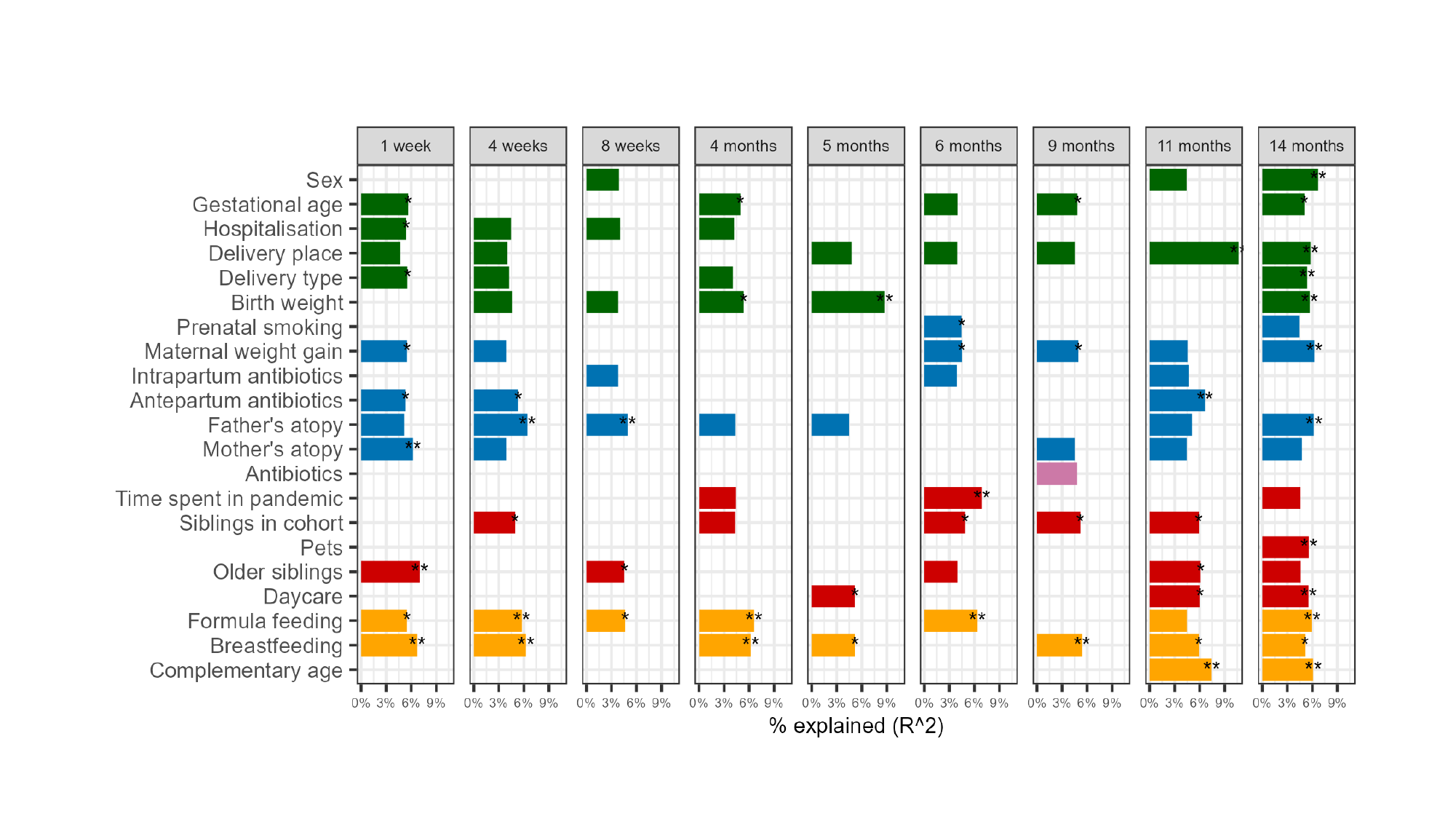

## Slide 17
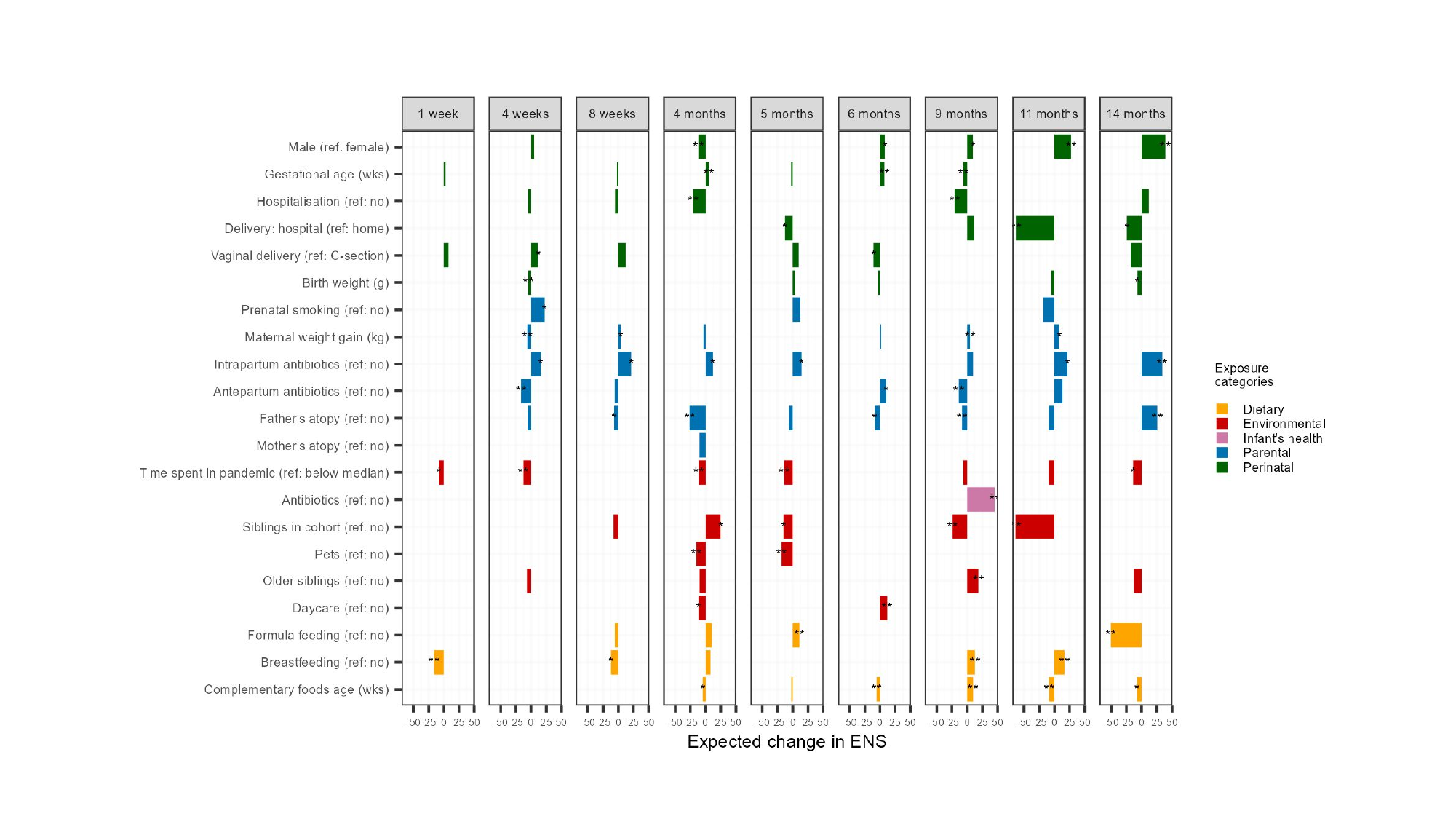

Supplement: Supplementary file 2 — Additional file 2: Supplementary Figures S1-S16. [file 13073_2026_1660_MOESM2_ESM.pptx]
